# Increased gene copy number of *DEFA1/DEFA3* worsens sepsis by inducing endothelial pyroptosis

**DOI:** 10.1073/pnas.1812947116

**Published:** 2019-02-04

**Authors:** QiXing Chen, Yang Yang, JinChao Hou, Qiang Shu, YiXuan Yin, WeiTao Fu, Feng Han, TingJun Hou, CongLi Zeng, Elizabeta Nemeth, Rose Linzmeier, Tomas Ganz, XiangMing Fang

**Affiliations:** ^a^Department of Clinical Research Center, Children’s Hospital, Zhejiang University School of Medicine, 310052 Hangzhou, China;; ^b^Department of Anesthesiology, The First Affiliated Hospital, Zhejiang University School of Medicine, 310003 Hangzhou, China;; ^c^College of Pharmaceutical Sciences, Zhejiang University, 310058 Hangzhou, China;; ^d^Institute of Functional Nano and Soft Materials, Soochow University, 215123 Suzhou, China;; ^e^Department of Medicine, David Geffen School of Medicine, University of California, Los Angeles, CA 90095

**Keywords:** sepsis, pyroptosis, endothelium, defensins, copy number polymorphism

## Abstract

Sepsis claims an estimated 30 million episodes and 6 million deaths per year, and treatment options are rather limited. Human neutrophil peptides 1–3 (HNP1–3) are the most abundant neutrophil granule proteins but their neutrophil content varies because of unusually extensive gene copy number polymorphism. A genetic association study found that increased copy number of the HNP-encoding gene *DEFA1/DEFA3* is a risk factor for organ dysfunction during sepsis development. However, direct experimental evidence demonstrating that these risk alleles are pathogenic for sepsis is lacking because the genes are present only in some primates and humans. Here, we generate *DEFA1/DEFA3* transgenic mice with neutrophil-specific expression of the peptides. We show that mice with high copy number of *DEFA1/DEFA3* genes have more severe sepsis-related vital organ damage and mortality than mice with low copy number of *DEFA1/DEFA3* or wild-type mice, resulting from more severe endothelial barrier dysfunction and endothelial cell pyroptosis after sepsis challenge. Mechanistically, HNP-1 induces endothelial cell pyroptosis via P2X7 receptor-mediating canonical caspase-1 activation in a NLRP3 inflammasome-dependent manner. Based on these findings, we engineered a monoclonal antibody against HNP-1 to block the interaction with P2X7 and found that the blocking antibody protected mice carrying high copy number of *DEFA1/DEFA3* from lethal sepsis. We thus demonstrate that *DEFA1/DEFA3* copy number variation strongly modulates sepsis development in vivo and explore a paradigm for the precision treatment of sepsis tailored by individual genetic information.

## Significance

Genetic copy number variations (CNVs) are the most common mechanism of structural genetic diversity, playing a key role in human health and common diseases. However, because of a lack of relevant experimental models, little is known about how rare CNVs contribute to the risk of complex phenotypes. We show that transgenic mice with high copy number of *DEFA1/DEFA3* [encoding human neutrophil peptides 1–3 (HNP1–3)] suffer from more severe sepsis because of more extensive endothelial barrier dysfunction and endothelial cell pyroptosis. Functional blockade of HNP1–3 rescues the mice from lethal sepsis. These findings exemplify how CNVs modulate disease development in appropriate animal models and explore a paradigm for the precision treatment of sepsis tailored by individual genetic information.

Sepsis is defined as a life-threatening organ dysfunction that is caused by a dysregulated host response to infection (1). It is a common disease state that occurs in many clinical contexts. Despite advances in intensive care management and goal-directed intervention, sepsis remains the leading cause of death among critically ill patients worldwide (2). Currently, there are no approved treatment options for sepsis because of an incomplete understanding of the key mechanisms regulating the host response to sepsis and its progression into organ dysfunction, in addition to the multifactorial nature of sepsis etiologies. Sepsis clearly imposes a substantial global burden of morbidity and mortality (3–5).

One of the most important pathophysiologic hallmarks in sepsis is the loss of the endothelial barrier function (6, 7). The endothelium is the largest organ in the body, composed of a highly dynamic cell layer that lines the interior surface of all blood vessels. The endothelium orchestrates a multitude of physiological functions, including the control of vascular tone, the movement of cells and nutrients, the maintenance of blood fluidity, and the growth of new vessels (8, 9). Loss of endothelial barrier function leads to the dysregulation of hemostasis and vascular reactivity, as well as tissue edema. Endothelial damage plays a central role in the progression to organ failure during sepsis and is a major contributor to sepsis mortality (10–12). More importantly, preserving endothelial barrier function has been shown to improve the outcome of sepsis (13–15). The mechanisms causing the quiescent endothelium to develop barrier dysfunction during sepsis might hold the key to future therapeutic strategies, but they are still largely unknown.

Defensins are short cationic, amphiphilic, cysteine-rich antimicrobial peptides with three or four intramolecular disulfide bonds. They are classified as α-, β-, and θ-defensins, of which the first two are the most common human antimicrobial peptides (16). The α-defensins, human neutrophil peptides (HNPs) 1–3, are constitutively expressed in neutrophils and are the most abundant neutrophil granule proteins. HNP1–3 differ in sequence in only the N-terminal amino acid, which is alanine for HNP-1 and aspartate for HNP-3. This amino acid is missing in HNP-2 peptide, and HNP-2 is thought to be a proteolytic product of HNP-1 and HNP-3 (16, 17). In addition to their broad repertoire of antimicrobial activities (18, 19), HNP1–3 exert multiple immunomodulatory effects (20–22), as well as early release alarmin activity to initiate the host response upon microbial invasion or tissue injury/damage (23). Studies in patients have documented that the levels of HNP1–3 in various body fluids (e.g., blood, bronchoalveolar lavage fluid, and sputum) are greatly increased during sepsis (24). Furthermore, our previous genetic association study found that the doses of genes encoding HNP1–3 (*DEFA1/DEFA3*), which display extensive copy number variations (CNVs) (25, 26), significantly impacted the clinical phenotype of sepsis, so that Chinese Han patients with high genetic copy number of *DEFA1/DEFA3* were more susceptible to severe sepsis (defined as sepsis plus organ dysfunction according to the old criteria for defining sepsis) (27). This association is not unique because CNVs have been suggested to play important roles in other complex diseases (28–30). However, despite the implication of HNP1–3 in sepsis outcomes, little is known about the functional role of *DEFA1/DEFA3* variants in the pathophysiology of sepsis. Experiments to analyze the specific roles of *DEFA1/DEFA3* CNVs in sepsis are challenging because mice naturally lack neutrophil defensins (16). Thus, we employed transgenic mice expressing HNP1–3 to explore the nonredundant functions of *DEFA1/DEFA3* genes in sepsis.

Here, we present experimental evidence for the impact of *DEFA1/DEFA3* CNVs on sepsis development and explore the mechanism by which HNP1–3 promotes endothelial barrier dysfunction and endothelial cell pyroptosis. We propose that functional blockade of HNP1–3 in a genetically tailored manner is rational strategy for development as a treatment for sepsis.

## Results

### Increased *DEFA1/DEFA3* Gene Dose Worsens Outcomes in Sepsis.

To investigate the potential role of genetic CNVs of *DEFA1/DEFA3* in sepsis development in vivo, we constructed a transgenic mouse model that expresses human *DEFA1/DEFA3* under its endogenous regulatory sequences to recapitulate human defensin pathophysiology. The mice were generated through microinjection of a P1 artificial chromosome construct (clone P1 34), including the human *DEFA1/DEFA3* genes into fertilized oocytes of C57BL/6 mice, and two independent *DEFA1/DEFA3* founder lines were obtained. The two founder lines carry 6 copies and 48 copies of *DEFA1/DEFA3* genes, and the transgenes have integrated into chromosome 5qF and chromosome 6qF3, respectively. The transgenic mice were viable and had normal growth, fertility, and lifespan. Immunohistochemical analysis confirmed the expression of the *DEFA1/DEFA3* genes in the bone marrow (BM) myeloid cells of transgenic mice and a cell-specific expression pattern of the *DEFA1/DEFA3* genes in peripheral blood neutrophils, but not in monocytes and lymphocytes or in other perfused peripheral organs/tissues (*SI Appendix*, Fig. S1*A*). Western blot analysis confirmed that BM myeloid cells of mice carrying more copies of *DEFA1/DEFA3* genes had much higher HNP protein levels than those of mice with lower copy number (*SI Appendix*, Fig. S1*B*). The two transgenic lines are referred to as mice with low copy numbers (LCN) and high copy numbers (HCN) of *DEFA1/DEFA3* genes, respectively, and are compared with WT mice for potential biological differences.

**Fig. 1.**
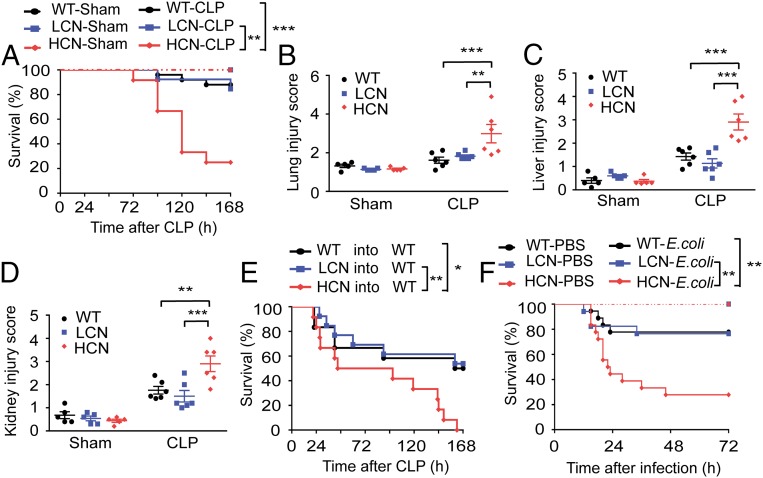
Increased gene copy number of *DEFA1/DEFA3* aggravates sepsis development. (*A*) Survival over time after performance of CLP in WT mice (*n* = 25), mice with a LCN of *DEFA1/DEFA3* (*n* = 13), and mice with a HCN of *DEFA1/DEFA3* (*n* = 12). Sham-operated mice were included as controls (*n* = 10 for each group). **P = 0.002, ***P < 0.001, log-rank test. (*B*) Quantification of the lung injury score based on H&E-stained lung sections of WT mice, mice with LCN of *DEFA1/DEFA3*, and mice with HCN of *DEFA1/DEFA3* at 72 h after CLP or sham operation. **P = 0.003, ***P < 0.001, two-way ANOVA with Bonferroni posttests. (*C*) Quantification of the liver injury score based on H&E-stained liver sections of WT mice, mice with LCN of *DEFA1/DEFA3*, and mice with HCN of *DEFA1/DEFA3* at 72 h after CLP or sham operation. ***P < 0.001, two-way ANOVA with Bonferroni posttests. (*D*) Quantification of the kidney injury score based on H&E-stained kidney sections of WT mice, mice with LCN of *DEFA1/DEFA3*, and mice with HCN of *DEFA1/DEFA3* at 72 h after CLP or sham operation. **P = 0.002, ***P < 0.001, two-way ANOVA with Bonferroni posttests. (*E*) Survival over time of lethally irradiated WT mice that were reconstituted with BM from WT mice (*n* = 12), mice with LCN of *DEFA1/DEFA3* (*n* = 13), and mice with HCN of *DEFA1/DEFA3* (*n* = 12) and that were subsequently subjected to CLP. *P = 0.016, **P = 0.006, log-rank test. (*F*) Survival over time after intraperitoneal infection of WT mice (*n* = 18), mice with LCN of *DEFA1/DEFA3* (*n* = 17), and mice with HCN of *DEFA1/DEFA3* (*n* = 18) with *E. coli* (4 × 10^6^ colony forming units per mouse) prepared in 200 μL of PBS. Mice intraperitoneally injected with 200 μL of PBS were included as controls (*n* = 10 for each group). **P = 0.004 between WT mice and mice with HCN of *DEFA1/DEFA3*, **P = 0.008 between mice with LCN of *DEFA1/DEFA3* and mice with HCN of *DEFA1/DEFA3*, log-rank test. In *B*–*D*, data are presented as dot plots with horizontal bars representing means ± SEM; *n* = 6 for each CLP group, and *n* = 5 for each sham group.

To study the impact of genetically increased *DEFA1/DEFA3* copy numbers on the development of sepsis, the transgenic mice and WT mice were subjected to sublethal cecal ligation and puncture (CLP), a commonly used sepsis model (31), and were monitored for survival and pathophysiological changes. Interestingly, 75% of the mice carrying HCN of *DEFA1/DEFA3* genes (9 of 12) died within 7 d after CLP, whereas only 15% of the mice with LCN of *DEFA1/DEFA3* genes (2 of 13) and 12% of the WT mice (3 of 25) died over the 7-d study period (Fig. 1*A*). Moreover, histopathologic analysis of lung (Fig. 1*B* and *SI Appendix*, Fig. S2*A*), liver (Fig. 1*C* and *SI Appendix*, Fig. S2*B*), and kidney (Fig. 1*D* and *SI Appendix*, Fig. S2*C*) revealed that, compared with the other two groups, the mice carrying HCN of *DEFA1/DEFA3* genes showed more severe organ injury, especially at 72 h after CLP. These data suggest that increased dosage of *DEFA1/DEFA3* genes in mice leads to a poor outcome of sepsis.

To confirm the contribution of cell-specific expression of the transgenes to the observed effect on sepsis, we generated BM-chimeric mice by reconstituting lethally irradiated WT mice with syngeneic BM with LCN of *DEFA1/DEFA3*, HCN of *DEFA1/DEFA3*, or WT. Notably, the chimeric mice receiving BM with HCN of *DEFA1/DEFA3* phenocopied the original transgenic mice with HCN of *DEFA1/DEFA3*. After induction of sepsis using sublethal CLP, HCN mice showed a significantly reduced survival rate compared with the other two groups (Fig. 1*E*).

To test the effect of increased dosage of *DEFA1/DEFA3* genes in another clinically relevant murine model of sepsis, we used intraperitoneal administration of *Escherichia coli*. Again, mice carrying HCN of *DEFA1/DEFA3* had a significantly higher mortality than mice with LCN of *DEFA1/DEFA3* and WT mice (Fig. 1*F*).

Thus, using the *DEFA1/DEFA3* transgenic mouse models, these findings suggest a causative link between *DEFA1/DEFA3* CNVs and sepsis outcome.

### Increased *DEFA1/DEFA3* Gene Dosage Leads to Endothelial Barrier Dysfunction During Sepsis.

Sepsis is a frequently fatal condition characterized by an uncontrolled and harmful host reaction to microbial infection (3, 4). HNP1–3 have multiple biological functions in host defense and inflammation, including antimicrobial action and the regulation of proinflammatory cytokine production (16, 17, 20). Therefore, we questioned whether increased *DEFA1/DEFA3* gene dosage could affect the host inflammatory response and bacterial clearance during sepsis. However, after sepsis onset, there were no significant differences among the three studied groups in bacterial burden in the peritoneal cavity and peripheral blood (*SI Appendix*, Fig. S3 *A* and *B*) or in plasma levels of inflammatory mediators TNF-α, IL-1β, IL-6, IL-10, IFN-γ, and IL-17A, as well as KC and MCP-1 (*SI Appendix*, Fig. S3 *C*–*J*). These results indicate that the detrimental effect of increased dosage of *DEFA1/DEFA3* genes on sepsis is not attributable to a dysregulated inflammatory or antimicrobial response.

**Fig. 2.**
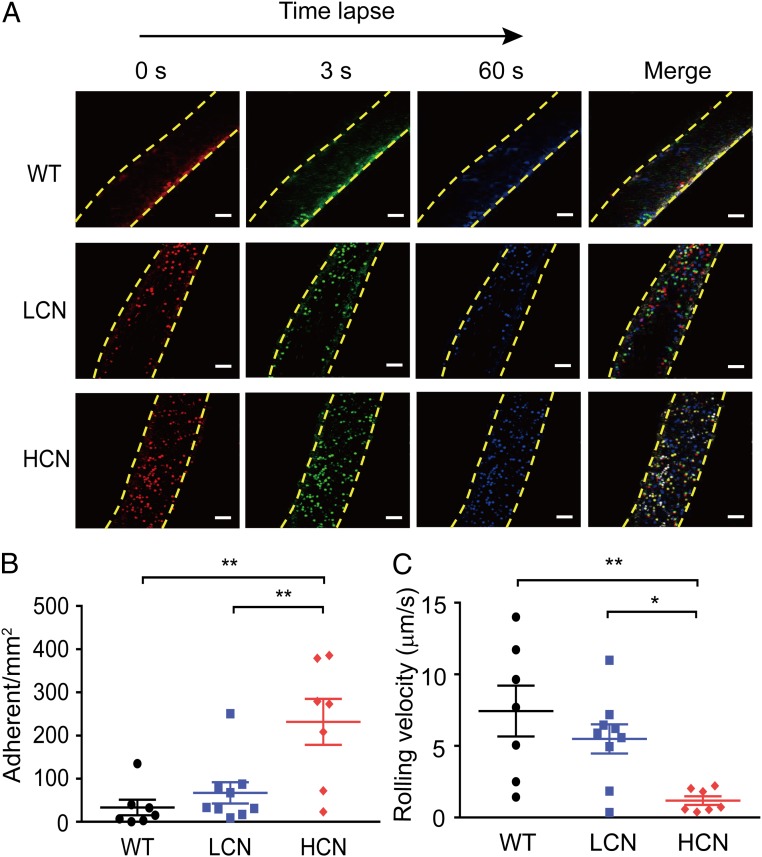
Increased gene copy number of *DEFA1/DEFA3* exacerbates leukocyte adhesion following sepsis. (*A*) Representative in vivo time-lapse imaging by two-photon laser scanning microscopy showing leukocyte adhesion in mesenteric vessels in WT mice, mice with LCN of *DEFA1/DEFA3*, and mice with HCN of *DEFA1/DEFA3* at 72 h after CLP. Images are individual frames from a continuous time-lapse movie. Times are presented in seconds. In each group, the first panel shows baseline adhesion in the vessel, the second and third panels show leukocyte adhesion at 3 s and 60 s after the baseline imaging, and the fourth panel shows the merged images. Red indicates leukocyte adhesion at baseline; green indicates leukocyte adhesion at 3 s after baseline; blue indicates leukocyte adhesion at 60 s after the baseline; yellow dashed lines indicate the vessel. (Scale bars, 50 μm.) (*B*) Quantitative measurements of firmly adhering leukocytes in WT mice, mice with LCN of *DEFA1/DEFA3*, and mice with HCN of *DEFA1/DEFA3* at 72 h after CLP. Firmly adherent leukocytes from at least three fields-of-view per mouse were counted as the number of cells per square millimeter of vascular surface area. **P = 0.002 between WT mice and mice with HCN of *DEFA1/DEFA3*, **P = 0.007 between mice with LCN of *DEFA1/DEFA3* and mice with HCN of *DEFA1/DEFA3*, one-way ANOVA with Bonferroni posttests. (*C*) Quantitative measurements of leukocyte rolling velocities in WT mice, mice with LCN of *DEFA1/DEFA3*, and mice with HCN of *DEFA1/DEFA3* at 72 h after CLP. *P = 0.048, **P = 0.005, one-way ANOVA with Bonferroni posttests. In *B* and *C*, *n* = 7 for WT mice with CLP and mice carrying HCN of *DEFA1/DEFA3* with CLP, *n* = 9 for mice carrying LCN of *DEFA1/DEFA3* with CLP.

Excessive endothelial activation and dysfunction constitutes a common host response to sepsis and is likely a key contributor to the pathogenesis of sepsis (10–12). We asked whether endothelial dysfunction played an important role in worse sepsis outcomes of mice with HCN of *DEFA1/DEFA3* genes. To address this issue, we first used two-photon intravital confocal microscopy to study leukocyte adhesion in a single mesenteric vessel in living mice at 72 h after CLP. Rhodamine 6G-labeled leukocytes were captured by sequential videos and images (Fig. 2*A* and Movies S1–S3), and they were quantified using time-lapse imaging. Adherent cells were defined as rhodamine 6G^+^ cells that remained stationary along the inner surface of the vessel wall for at least 1 min. Interestingly, the number of firmly adherent leukocytes in mice carrying HCN of *DEFA1/DEFA3* at 72 h post-CLP was 285 ± 39/mm^2^, which was significantly higher than that in mice with LCN of *DEFA1/DEFA3* and in WT mice (129 ± 27/mm^2^ and 34 ± 13/mm^2^, respectively) (Fig. 2*B*). In contrast, leukocytes from the related sham-operated controls exhibited little adherence to the microvasculature (*SI Appendix*, Fig. S4 *A* and *B* and Movies S4–S6). In agreement with leukocyte adhesion findings, a significant reduction in rolling velocity in the mesenteric vessels of septic mice carrying HCN of *DEFA1/DEFA3* was also observed (Fig. 2*C* vs. *SI Appendix*, Fig. S4*C*). These findings provide important in vivo evidence for aggregated leukocyte adhesion and suggest a possible role of endothelial activation in the progression of sepsis in mice carrying HCN of *DEFA1/DEFA3*.

Because endothelial activation is an early step in sepsis, we explored if expression of endothelial adhesion molecules in vital organs increased under the condition of sepsis. Immunohistochemistry revealed a low degree of intercellular adhesion protein 1 (ICAM-1) and vascular cell adhesion protein 1 (VCAM-1) expression in lungs of WT mice and mice with LCN of *DEFA1/DEFA3*, but a significantly increased expression of ICAM-1 and VCAM-1 in lungs of mice carrying HCN of *DEFA1/DEFA3*, at 24 h after sepsis onset (*SI Appendix*, Fig. S5 *A*–*D*). We also investigated whether there were any changes in expression of leukocyte adhesion molecules, and found that the expression levels of lymphocyte function associated antigen-1 (LFA-1) and very late antigen-4 (VLA-4) in peripheral blood leukocytes were very low and not significantly different among the three genotype groups (*SI Appendix*, Fig. S5 *E* and *F*).

Neutrophil-borne HNP-1 can synergize with platelets to stimulate monocyte adhesion and enhance their recruitment, hence participating in acute and chronic inflammation (21). Combined with our results, we tested whether the observed phenotype in transgenic mice after sepsis challenge could be attributed to an altered monocyte function. Thus, monocytes/macrophages were depleted 48 h before CLP performance using clodronate liposomes. Notably, the depletion of monocytes/macrophages did not impact the survival of mice carrying HCN of *DEFA1/DEFA3* (*SI Appendix*, Fig. S6).

**Fig. 3.**
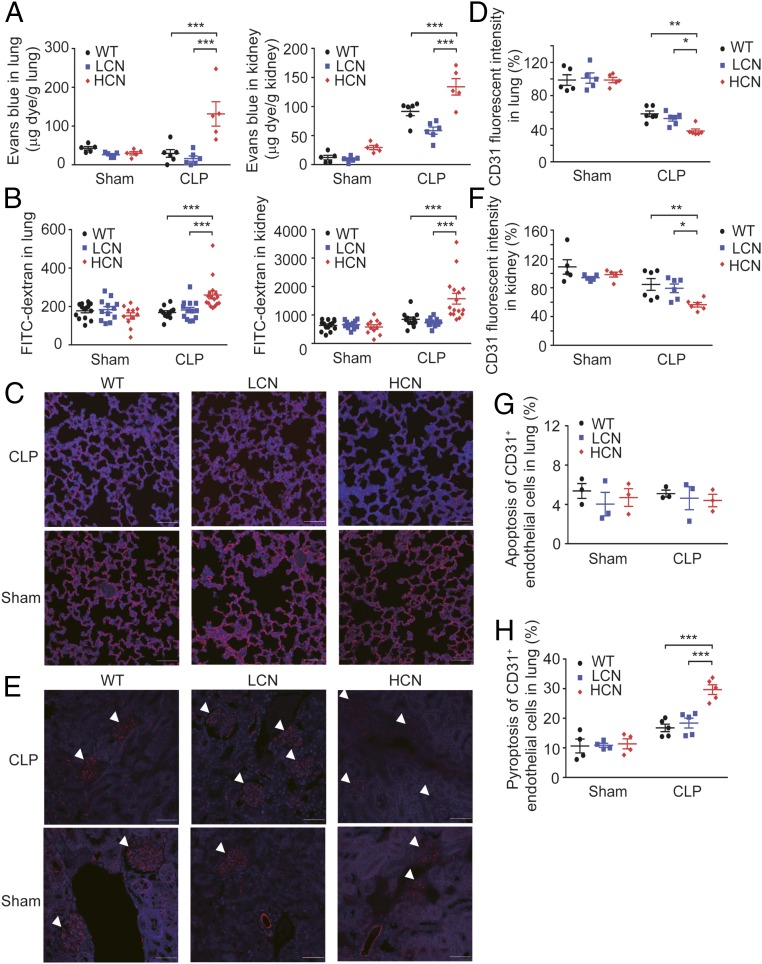
Increased gene copy number of *DEFA1/DEFA3* leads to endothelial barrier dysfunction and endothelial cell pyroptosis after sepsis. (*A*) In vivo Evans blue dye permeability assay in lungs (*Left*) and kidneys (*Right*) at 72 h after performance of CLP or sham operation in WT mice (*n* = 6 for CLP, and *n* = 5 for sham), mice with LCN of *DEFA1/DEFA3* (*n* = 6 for CLP, and *n* = 5 for sham), and mice with HCN of *DEFA1/DEFA3* (*n* = 5 for each CLP and sham group). ***P < 0.001, two-way ANOVA with Bonferroni posttests. (*B*) In vivo FITC-dextran permeability assay in lungs (*Left*) and kidneys (*Right*) at 72 h after performance of CLP or sham operation in WT mice (*n* = 11 for CLP, and *n* = 14 for sham), mice with LCN of *DEFA1/DEFA3* (*n* = 13 for CLP, and *n* = 12 for sham), and mice with HCN of *DEFA1/DEFA3* (*n* = 16 for CLP, and *n* = 11 for sham). ***P < 0.001, two-way ANOVA with Bonferroni posttests. (*C*–*F*) Representative images and comparisons of CD31^+^ endothelial cells in the lung (*C* and *D*) and renal glomeruli (*E* and *F*) at 72 h after performance of CLP or sham operation in WT mice (*n* = 6 for CLP, and *n* = 5 for sham), mice with LCN of *DEFA1/DEFA3* (*n* = 6 for CLP, and *n* = 5 for sham), and mice with HCN of *DEFA1/DEFA3* (*n* = 6 for CLP, and *n* = 5 for sham). The white arrowheads in *E* indicate renal glomeruli. CD31^+^ endothelial cells were quantified by the fluorescence intensity in at least five different high-power fields from each slide. Red indicates CD31 staining; blue indicates DAPI staining. (Scale bars, 50 μm.) In *D*, *P = 0.040, **P = 0.003, two-way ANOVA with Bonferroni posttests. In *F*, *P = 0.034, **P = 0.007, two-way ANOVA with Bonferroni posttests. (*G*) Quantification of apoptotic endothelial cells in the lung at 48 h after performance of CLP or sham operation in WT mice (*n* = 3 for each CLP and sham group), mice with LCN of *DEFA1/DEFA3* (*n* = 3 for each CLP and sham group), and mice with HCN of *DEFA1/DEFA3* (*n* = 3 for each CLP and sham group) using flow cytometry. See also *SI Appendix*, Fig. S8*A* for flow cytometry gating strategy. (*H*) Quantification of pyroptotic endothelial cells in the lung at 48 h after performance of CLP or sham operation in WT mice (*n* = 5 for CLP, and *n* = 4 for sham), mice with LCN of *DEFA1/DEFA3* (*n* = 5 for CLP, and *n* = 4 for sham), and mice with HCN of *DEFA1/DEFA3* (*n* = 5 for CLP, and *n* = 4 for sham) using flow cytometry. See also *SI Appendix*, Fig. S8*B* for flow cytometry gating strategy. ***P < 0.001, two-way ANOVA with Bonferroni posttests. In *A*, *B*, *D*, and *F*–*H*, data are presented as dot plots with horizontal bars representing means ± SEM.

To further examine the contribution of endothelial activation and dysfunction to the pathogenesis of sepsis in transgenic mice, the effects of these genetic variations on vascular endothelial integrity in vital organs/tissues, such as lungs, kidneys, and mesenteric vessel beds were studied. Evans blue dye and FITC-conjugated dextran leakage analyses are commonly used to assess endothelial integrity and vascular permeability in vivo. In both assays, compared with WT mice and the mice with LCN, those carrying HCN of *DEFA1/DEFA3* had significantly increased extravasation of dyes in the vessel beds of lungs, kidneys, and mesentery at 72 h after the initiation of sepsis (Fig. 3 *A* and *B* and *SI Appendix*, Fig. S7 *A* and *B*). Taken together, these data show that endothelial barrier dysfunction contributes to sepsis deterioration in mice carrying HCN of *DEFA1/DEFA3* genes.

Taken together, these findings demonstrate that after sepsis onset, vascular endothelia are excessively activated and dysfunctional in mice carrying HCN of *DEFA1/DEFA3*.

### Increased *DEFA1/DEFA3* Gene Dose Results in Endothelial Cell Pyroptosis During Sepsis.

To characterize how endothelial barrier function was affected in mice with HCN of *DEFA1/DEFA3*, we then examined whether the endothelial cells were directly damaged during septic challenge. CD31 is a surface marker expressed by endothelial cells (32). Using immunofluorescence analysis, we found that the sham-operated mice from the three studied groups preserved intact endothelial cells in pulmonary, renal, and mesenteric microvessels. Notably, at 72 h after sepsis development, the mice carrying HCN of *DEFA1/DEFA3* showed a marked loss of CD31^+^ endothelial cells in microvessels from lung (Fig. 3 *C* and *D*), kidney (Fig. 3 *E* and *F*), and mesentery (*SI Appendix*, Fig. S7 *C* and *D*), which presumably resulted from lethal injury of the endothelial cells.

Endothelial cell loss resulting from cell death has been observed during severe infections and inflammation and been demonstrated to contribute to vascular permeability (10). We next examined whether endothelial cell death was responsible for more severe endothelial barrier dysfunction in septic mice carrying the *DEFA1/DEFA3* HCN variant. Single-cell suspension that includes endothelial cells was generated from mouse lung and analyzed using flow cytometry. Both in transgenic mice and in WT mice, only a small proportion of CD31^+^ endothelial cells exhibited apoptotic characteristics at 48 h after sepsis induction (*SI Appendix*, Fig. S8*A*). In addition, the endothelial cells from the three grouped mice showed no significant difference in apoptosis (Fig. 3*G*). We therefore explored whether these cells underwent pyroptosis, a type of inflammatory cell death that has recently been found to contribute endothelial barrier dysfunction in infectious and inflammatory diseases (33, 34). Strikingly, a higher proportion of CD31^+^ endothelial cells showed pyroptotic death [stained with propidium iodide (PI) and fluorescent-labeled inhibitors of caspase-1 (FLICA)–caspase-1, a key player in pyroptosis (35)] at 48 h after sepsis onset (*SI Appendix*, Fig. S8*B*). Moreover, this phenomenon was particularly pronounced in mice carrying HCN of *DEFA1/DEFA3* (Fig. 3*H*). Thus, these data provide evidence of enhanced endothelial cell pyroptosis in sepsis pathogenesis in mice carrying the *DEFA1/DEFA3* risk variant.

### HNP-1 Promotes Endothelial Cell Pyroptosis.

**Fig. 4.**
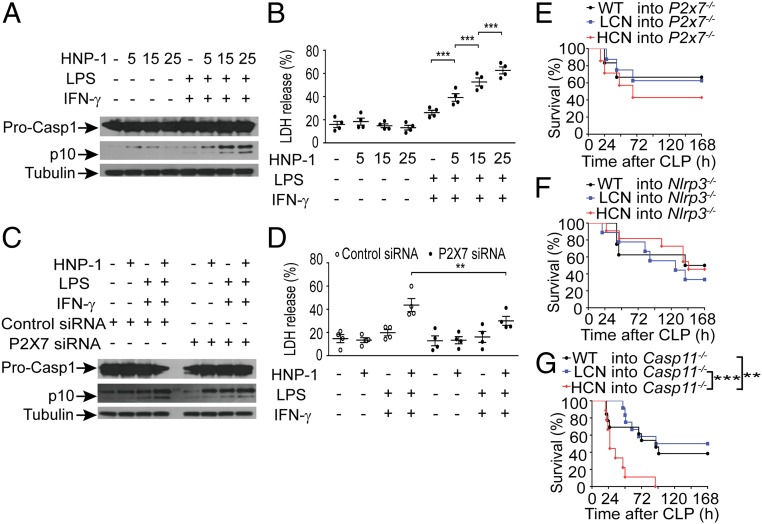
HNP-1 induces endothelial cell pyroptosis through P2X7-mediating canonical caspase-1 activation. (*A*) Representative Western blot analysis of caspase-1 precursor (Pro-CASP1) and mature caspase-1 (p10) following stimulation with different concentrations of HNP-1 (0 μg/mL, 5 μg/mL, 15 μg/mL, 25 μg/mL) in MLMECs that were already primed with LPS (1 μg/mL) and IFN-γ (100 ng/mL). One representative of four independent experiments is shown. α-Tubulin served as a loading control. See also *SI Appendix*, Fig. S16 for uncropped images of immunoblots. (*B*) Cell toxicity (measured by LDH release into the medium) in response to different concentrations of HNP-1–stimulated MLMECs that were already primed with LPS and IFN-γ (*n* = 4). ***P < 0.001, one-way ANOVA with Bonferroni posttests. (*C*) Representative Western blot analysis of Pro-CASP1 and p10 caspase-1 following HNP-1 (25 μg/mL) stimulation in P2X7 siRNA and control siRNA-transfected MLMECs that were already primed with LPS and IFN-γ. One representative of four independent experiments is shown. α-Tubulin served as a loading control. See also *SI Appendix*, Fig. S16 for uncropped images of immunoblots. (*D*) Cell toxicity (measured by LDH release into the medium) following HNP-1 (25 μg/mL) stimulation in P2X7 siRNA and control siRNA-transfected MLMECs that were already primed with LPS and IFN-γ (*n* = 4). **P = 0.004, one-way ANOVA with Bonferroni posttests. (*E*–*G*) Survival over time of lethally irradiated *P2x7*^*−/−*^ mice (*E*), *NLRP3*^*−/−*^ mice (*F*), and *caspase-11*^*−/−*^ mice (*G*) that were reconstituted with BM from WT mice, mice with LCN of *DEFA1/DEFA3*, and mice with HCN of *DEFA1/DEFA3* and that were subsequently subjected to CLP. In *E*, *n* = 6 for mice reconstituted with BM from WT mice, *n* = 8 for mice reconstituted with BM from mice carrying LCN of *DEFA1/DEFA3*, and *n* = 7 for mice reconstituted with BM from mice carrying HCN of *DEFA1/DEFA3*. In *F*, *n* = 8 for mice reconstituted with BM from WT mice, *n* = 9 for mice reconstituted with BM from mice carrying LCN of *DEFA1/DEFA3*, and *n* = 11 for mice reconstituted with BM from mice carrying HCN of *DEFA1/DEFA3*. In *G*, *n* = 13 for mice reconstituted with BM from WT mice, *n* = 12 for mice reconstituted with BM from mice carrying LCN of *DEFA1/DEFA3*, and *n* = 9 for mice reconstituted with BM from mice carrying HCN of *DEFA1/DEFA3*. **P = 0.009, ***P < 0.001, log-rank test. In *B* and *D*, data are presented as dot plots with horizontal bars representing means ± SEM.

To determine how the increased dose of *DEFA1/DEFA3* led to endothelial cell pyroptosis during sepsis, we first examined the protein level of HNP1–3 in vivo after septic challenge. Immunohistochemical analysis of lung tissues from WT and transgenic mice revealed markedly elevated levels of HNP1–3 in mice carrying HCN of *DEFA1/DEFA3* at 24 and 48 h after CLP (*SI Appendix*, Fig. S9). Consequently, we focused our further investigations on the impact of HNP1–3 on endothelial cell viability. We assessed the direct effect of HNP-1 on endothelial cell pyroptosis in vitro using a mouse lung microvascular endothelial cell (MLMEC) line. Interestingly, we found that a higher concentration of HNP-1 could activate more cleaved caspase-1 in primed MLMECs (Fig. 4*A*). In addition, a higher concentration of HNP-1 promoted more cell death, as reflected by lactate dehydrogenase (LDH) release (Fig. 4*B*). However, two other important mediators of pyroptosis, noncanonical caspase-11 inflammasome and gasdermin D (36, 37), were not activated (*SI Appendix*, Fig. S10 *A* and *B*). Cleaved caspase-3 was also not detected (*SI Appendix*, Fig. S10*C*). These dose-dependent effects of HNP-1 on endothelial cell viability were closely consistent with in vivo findings showing that mice carrying HCN of *DEFA1/DEFA3* had more pyroptotic endothelial cells than those with LCN of *DEFA1/DEFA3* and WT controls during sepsis development.

HNP1–3 has been reported to play multifunctional roles in the immune system, but the specific receptors related to these activities have not yet been well characterized. Our previous study identified HNP-1 through direct binding to the purinergic receptor P2X ligand-gated ion channel 7 (P2X7), promoting LPS-primed macrophage pyroptosis (38). We therefore examined whether this receptor is responsible for HNP1–3-induced pyroptosis in endothelial cells. Both in vivo and in vitro studies showed that the expression levels of P2X7 in endothelial cells were markedly increased under septic and inflammatory conditions (*SI Appendix*, Figs. S11 and S12), suggesting a potential function of P2X7. Furthermore, knockdown of P2X7 in primed MLMECs using small interference RNA dramatically reduced HNP-1–promoted cleaved caspase-1 levels as well as released LDH levels (Fig. 4 *C* and *D*). These results show that HNP-1 activates caspase-1 inflammasome to induce pyroptotic death in primed endothelial cells via interaction with P2X7.

To further examine whether such a mechanism contributes to the deleterious effect of increased dose of *DEFA1/DEFA3* on sepsis, BM-chimeric mice were generated by reconstituting lethally irradiated *P2X7*-deficient mice with BM from HCN of *DEFA1/DEFA3*, LCN of *DEFA1/DEFA3*, or WT mice. As expected, no significant difference in the survival rate was observed among the chimeric *P2X7*-deficient mice during the 7-d period after sepsis initiation (Fig. 4*E*). Because the Nod-like receptor family pyrin domain containing 3 (NLRP3) is characterized primarily as a canonical caspase-1–activating inflammasome downstream of P2X7 during pyroptosis (38–40), we also confirmed the role of NLRP3 inflammasome in the observed effect using BM-chimeric mice. The *Nlrp3*-deficient mice reconstituted with BM from HCN of *DEFA1/DEFA3*, LCN of *DEFA1/DEFA3*, or WT mice showed no significant difference in survival rate after sepsis onset (Fig. 4*F*). However, when chimeric *caspase-11*–deficient mice transplanted with BM from HCN of *DEFA1/DEFA3*, LCN of *DEFA1/DEFA3*, or WT mice were challenged with CLP, significantly higher mortality was observed in those transplanted with BM from mice with HCN of *DEFA1/DEFA3* (Fig. 4*G*). This in vivo observation is consistent with the in vitro findings showing that HNP-1 induces endothelial cell pyroptosis in a caspase-11–independent manner. Collectively, these data demonstrate that increased *DEFA1/DEFA3* gene dose, by producing elevated levels of HNP1–3, promotes endothelial cell pyroptosis and vascular leakage, which is dependent on P2X7-mediated canonical NLRP3/caspase-1 inflammasome activation.

### Blocking the Interaction of HNP1–3 and P2X7 Rescues Mice Carrying the *DEFA1/DEFA3* Risk Variant from Lethal Sepsis.

**Fig. 5.**
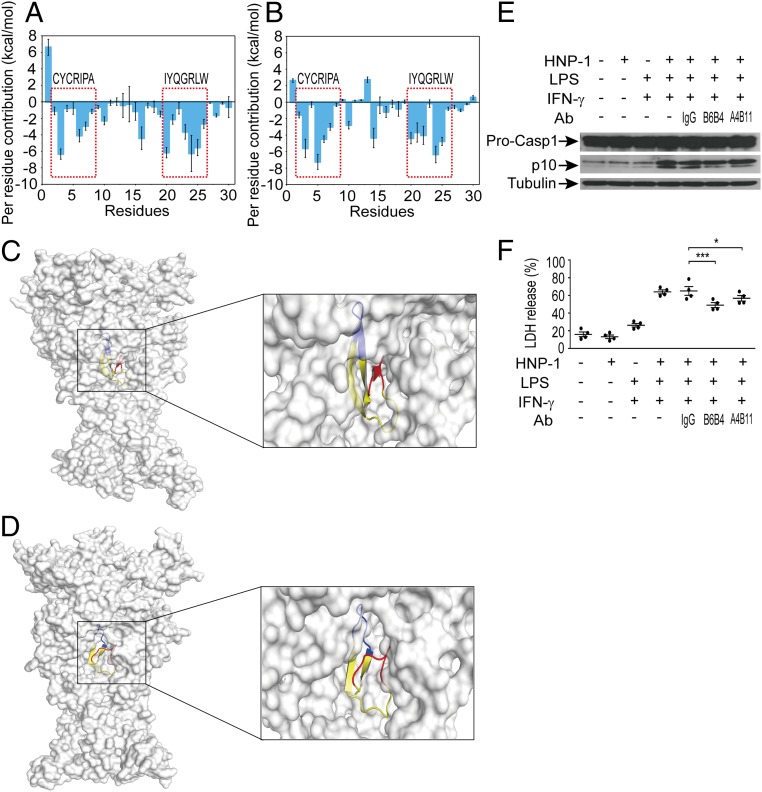
Monoclonal antibodies block the interaction between HNP-1 and P2X7 in vitro. (*A* and *B*) Per residue contributors to the binding free energy of HNP-1 with hP2X7 (*A*) or mP2X7 (*B*). (*C* and *D*) Structure analysis of the last snapshot of the HNP-1/hP2X7 complex (*C*) and HNP-1/mP2X7 complex (*D*) from MD simulations. The molecular surface views are presented. The three monomers of hP2X7 or mP2X7 are colored in gray. The CYCRIPA motif, IYQGRLW motif and other motifs of HNP-1 are colored in red, blue, and yellow, respectively. (*E*) Representative Western blot analysis of Pro-CASP1 and p10 caspase-1 following HNP-1 (25 μg/mL) combined with monoclonal antibody (B6B4 or A4B11) or control IgG treatment in MLMECs that were already primed by LPS (1 μg/mL) and IFN-γ (100 ng/mL). One representative of four independent experiments is shown. α-Tubulin served as a loading control. See also *SI Appendix*, Fig. S16 for uncropped images of immunoblots. (*F*) Cell toxicity (measured by LDH release into the medium) in HNP-1 combined with monoclonal antibody (B6B4 or A4B11) or control IgG-treated MLMECs that were already primed with LPS and IFN-γ (*n* = 4). Data are presented as dot plots with horizontal bars representing means ± SEM. *P = 0.028, ***P < 0.001, one-way ANOVA with Bonferroni posttests.

Based on these data, we reasoned that impeding the interaction between HNP1–3 and P2X7 might inactivate downstream signaling and protect the viability of endothelial cells. We therefore developed an antibody to block HNP1–3 recognition by P2X7 receptor. To this end, we used homologous modeling, molecular docking, and molecular dynamics (MD) simulation techniques to predict a likely heteromeric complex structure of HNP-1 bound to human P2X7 (hP2X7) or mouse P2X7 (mP2X7). The disallowed regions in the Ramachandran plots for both the modeled hP2X7 and mP2X7 were lower than 1% (hP2X7, 0.7%; mP2X7, 0.4%) (*SI Appendix*, Fig. S13 *A* and *B*), suggesting that the modeled structures were acceptable for further protein–protein docking calculations. For the HNP-1/hP2X7 or HNP-1/mP2X7 complex, a total of 3,600 structures were generated by the ZDOCK web server (41). The top 10 ranked binding structures for HNP-1/hP2X7 or HNP-1/mP2X7 showed high similarity (*SI Appendix*, Fig. S13 *C* and *D*). Therefore, the most stable structures generated by ZDOCK were used for the MD simulations. To monitor the stability of the simulated systems, the RMSDs of the C_α_ atoms from their initial position were analyzed. The RMSD values of hP2X7 and mP2X7 tended to converge after 30–45 ns of MD simulations (*SI Appendix*, Fig. S13 *E* and *F*). In addition, the RMSD values of HNP-1 in both the HNP-1/hP2X7 and HNP-1/mP2X7 complexes were quite stable during the simulations. Subsequently, the trajectories of the last 20-ns simulations were taken for the following energetic analysis. To illuminate the roles of individual residues in determining the interactions between HNP-1 and hP2X7 or mP2X7, the molecular mechanics/generalized born surface area (MM/GBSA) binding free energy was decomposed into the contributions of each residue. The results showed that the CYCRIPA and IYQGRLW motifs in HNP-1 formed favorable interactions with hP2X7 or mP2X7 (Fig. 5 *A* and *B*). In particular, the CYCRIPA motif may maintain the open state to allow for the transportation of ions in both HNP-1/hP2X7 and HNP-1/mP2X7 complexes (Fig. 5 *C* and *D* and *SI Appendix*, Fig. S13 *G* and *H*). Thus, the CYCRIPA motif was selected as an antigen to develop antibodies with the potential to inhibit the interaction between HNP-1 and P2X7.

Accordingly, we generated a set of monoclonal antibodies and screened them for their ability to bind HNP-1. Finally, two monoclonal antibodies (designated B6B4 and A4B11) were selected because of their cross-reactivity with HNP-1 [dissociation constant (*K*_*D*_) value of 40.2 nM for B6B4 and 13.8 nM for A4B11]. The antibodies were then tested to assess their ability to block HNP-1 binding to P2X7 on primed MLMECs. Preincubation of HNP-1 with the B6B4 antibody more significantly alleviated endothelial cell pyroptosis, as manifested by a markedly reduced level of activated caspase-1 and released LDH in primed MLMECs (Fig. 5 *E* and *F*), suggesting a potent interference with the HNP-1/P2X7 interaction.

**Fig. 6.**
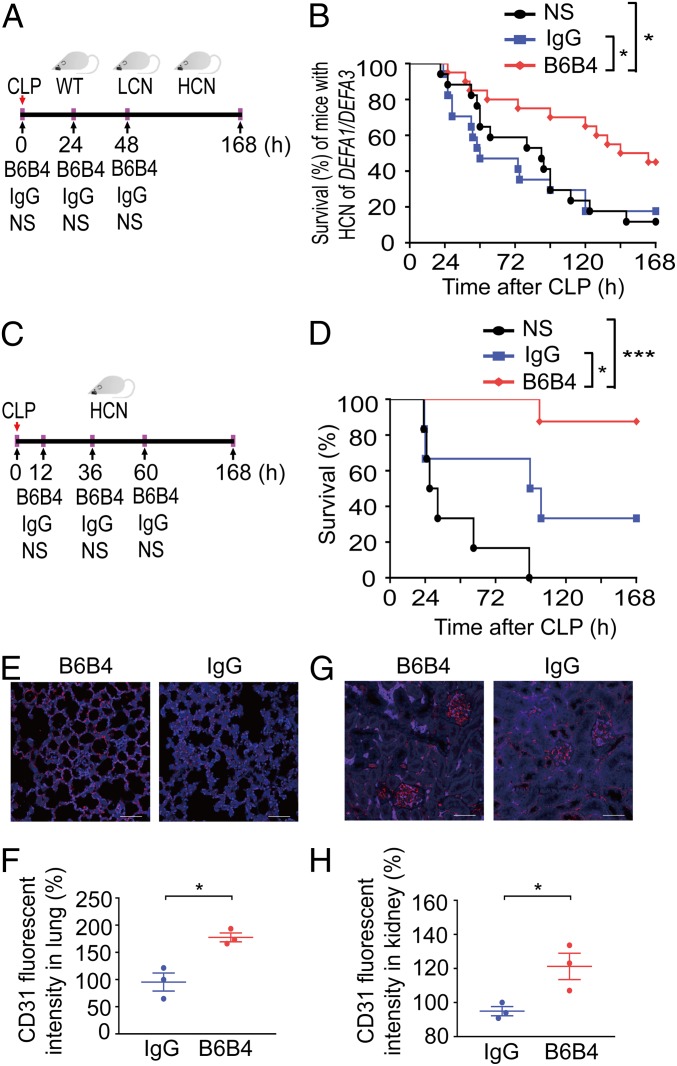
The blocking antibody B6B4 rescues mice with HCN of *DEFA1/DEFA3* from sepsis. (*A* and *B*) Mice were subjected to CLP, and B6B4 (300 μg per mouse), mouse IgG (300 μg per mouse), or normal saline (NS; equal volume) were intravenously administered at 0, 24, and 48 h after CLP (*A*). Survival over time of mice with HCN of *DEFA1/DEFA3* was assessed (*B*). *P = 0.011 between NS-treated group and B6B4-treated group, *P = 0.016 between IgG-treated group and B6B4-treated group, log-rank test. (*C* and *D*) Mice were subjected to CLP, and B6B4 (300 μg per mouse), mouse IgG (300 μg per mouse), or normal saline (NS; equal volume) were intravenously administered at 12, 36, and 60 h after CLP (*C*). Survival over time of mice with HCN of *DEFA1/DEFA3* was assessed (*D*). *P = 0.031, ***P < 0.001, log-rank test. (*E*–*H*) Representative images and comparisons of CD31^+^ endothelial cells in the lung (*E* and *F*) and renal glomeruli (*G* and *H*) from mice with HCN of *DEFA1/DEFA3* that were subjected to CLP and treated with mouse IgG (*n* = 3) or B6B4 (*n* = 3) as shown in *C*, and the mice were killed at 72 h after CLP. Red indicates CD31^+^ staining; blue indicates DAPI staining. (Scale bars, 50 μm.) In *B*, *n* = 17 for mice with NS treatment, *n* = 17 for mice with IgG treatment, and *n* = 20 for mice with B6B4 treatment. In *D*, *n* = 6 for mice with NS treatment, *n* = 6 for mice with IgG treatment, and *n* = 8 for mice with B6B4 treatment. In *F* and *H*, data are presented as dot plots with horizontal bars representing means ± SEM. In *F*, *P = 0.011, two-tailed unpaired *t* test. In *H*, *P = 0.032, two-tailed unpaired *t* test.

We next investigated the bioactivity of the blocking activity of B6B4 in vivo. We subjected mice carrying the HCN of *DEFA1/DEFA3* genes to the CLP model and treated them three times with the blocking antibody (300 μg, by tail vein injection; immediately, 24 and 48 h after performance of sepsis), or administered an equal amount of mouse IgG or an equal volume of normal saline (Fig. 6*A*). Notably, treatment with B6B4 antibody had a significant protective effect on septic mice carrying HCN of *DEFA1/DEFA3* genes, as evidenced by a reduced 7-d mortality (Fig. 6*B*). Additionally, mice treated with the B6B4 antibody generally appeared healthy, whereas those treated with IgG or normal saline were inactive and looked ill at 24 h after CLP (Movie S7). However, treatment with B6B4 antibody did not confer any protective effect on septic mice with LCN of *DEFA1/DEFA3* genes or with the WT genotype (*SI Appendix*, Fig. S14). To examine the therapeutic potential of the blocking antibody in established sepsis, we treated mice with HCN of *DEFA1/DEFA3* at an initial time of 12 h post-CLP and thereafter at 36 and 60 h (Fig. 6*C*). Strikingly, this treatment regimen more effectively protected mice with HCN of *DEFA1/DEFA3* from mortality (Fig. 6*D*). Immunofluorescent analysis showed substantially increased CD31^+^ endothelial cells in the lungs (Fig. 6 *E* and *F*) and kidneys (Fig. 6 *G* and *H*), as well as in mesenteric vessels (*SI Appendix*, Fig. S15), in B6B4 antibody-treated mice at 72 h after CLP, compared with IgG-treated controls. Thus, the blocking antibody against the interaction between HNP-1 and P2X7 can protect mice with HCN of *DEFA1/DEFA3* from lethal sepsis.

## Discussion

CNVs are the most common mechanism of structural genetic diversity and play a key role in human health and common diseases (42, 43). Although there is abundant evidence of beneficial duplications, particularly in the context of stressful or perturbed environmental conditions, increases in gene copy number are usually implicated in increased susceptibility to a wide range of human diseases (44). However, little is known about how rare CNVs contribute to the risk of the diseases and the complex phenotypes. Animal model experiments are the mainstay of distinguishing causality from association in human genetics. The use of transgenic mouse models allows for the study of direct effects of different gene dosages on specific phenotypes at the pathophysiological, cellular, and molecular levels (45, 46). In the present study, mice engineered to have HCN of *DEFA1/DEFA3* genes showed more severe organ damage and a worse outcome after sepsis challenge, indicating a genotype-restricted function in phenotype development. Thus, combined with our previous case-control genetic association study, these human and transgenic mouse model-based findings indicate that HCN of *DEFA1/DEFA3* is a causative genetic factor for sepsis development.

Defensins contribute to innate immunity through diverse actions. Neutrophil-derived α-defensins are able to affect profound changes in the inflammatory environment while also serving as effective antimicrobial peptides. HNP-1 functions as a molecular brake on macrophage-driven inflammation by preventing protein translation (22). However, no pathophysiological differences related to these biological activities of HNP1–3 were detected between HCN and LCN of *DEFA1/DEFA3* mice during sepsis development. Interestingly, a recent study by London et al. (13) also found that activating an endothelium-specific, Robo4-dependent signaling pathway with the soluble ligand Slit could strengthen the vascular barrier, reduce vascular permeability in the lung and other organs, and increase survival in different murine models of sepsis, but did not alter plasma concentrations of a panel of cytokines and chemokines. These findings indicate that the marked and abrupt cytokine storm during sepsis is mainly generated by the innate immune response triggered by microbial infection or initial insult. We surmise that vascular endothelial dysfunction can result in severe tissue/organ damage and fatal outcome, without further amplifying the inflammatory response during sepsis. Thus, the breakdown in endothelial barrier function plays a crucial role in the pathogenesis of sepsis, and buttressing the endothelial barrier may be sufficient to reduce mortality from sepsis.

Emerging evidence suggests that endothelial activation in vital organ vasculatures is an early and key step in the pathogenesis of sepsis. Endothelial cell activation is marked by the expression of endothelial cell-surface adhesion molecules, such as VCAM-1, and ICAM-1 (47). In the present study, significantly elevated expression levels of VCAM-1 and ICAM-1 were observed in the lung tissue of mice carrying HCN of *DEFA1/DEFA3* at 24 h after sepsis onset compared with those of mice with LCN of *DEFA1/DEFA3* and WT mice. These findings indicate a more activated state of vascular endothelial cells in vital organs of mice carrying HCN of *DEFA1/DEFA3* after sepsis challenge. Excessive endothelial activation can lead to endothelial dysfunction with the expression as endothelial cell death. On the other hand, neutrophil HNP-1 can stimulate monocyte adhesion and enhance their recruitment to participate in acute and chronic inflammation (21). However, depletion of macrophage/monocytes did not confer any protection against lethal sepsis for mice carrying HCN of *DEFA1/DEFA3*. Thus, mechanisms such as endothelial cell death are favored to contribute to the endothelial barrier dysfunction in septic mice carrying HCN of *DEFA1/DEFA3*.

Our in vivo results strongly suggest a connection between the *DEFA1/DEFA3* risk variant and the breakdown of endothelial barrier function. Endothelial barrier dysfunction is a central pathogenic feature of sepsis. There is increasing evidence that widespread endothelial cell death, especially pyroptotic cell death, is an initial mechanism mediating endothelial injury under inflammatory and infectious conditions. For example, caspase-11–mediated endothelial pyroptosis is requisite for endotoxemia-induced lung injury (33), while endothelial cell pyroptosis via caspase-1–dependent inflammasome activation leads to endothelial dysfunction in hyperhomocysteinemia-related vascular inflammation and atherosclerosis (34). In the current study, caspase-1–dependent, but not caspase-11–mediated, endothelial pyroptosis was found both in LPS-primed endothelial cells stimulated by HNP-1 and in the *DEFA1/DEFA3* risk-variant transgenic mice after sepsis progression, where HNP-1–dependent damage was triggered through signaling by P2X7 receptor. In agreement with these findings, the BM chimera experiments further affirmed a central role for nonhematopoietic P2X7 and its downstream inflammasome components NLRP3 and caspase-1 in the pathogenic changes. These data highlight the importance of endothelial cell survival and endothelial barrier function during sepsis challenge in *DEFA1/DEFA3* risk-variant transgenic mice.

Importantly, mechanistic insights into the interaction between HNP-1 and P2X7 raised the possibility of new therapeutic modalities that we could test in septic mice carrying the *DEFA1/DEFA3* risk variant. Based on the structural model developed in the present study, we generated a blocking antibody to impede the binding of HNP1–3 to P2X7. Treatment with this monoclonal antibody in the *DEFA1/DEFA3* risk-variant transgenic mice, but not in the mice with other genotypes, indeed dramatically improved endothelial barrier function and protected mice from sublethal sepsis, suggesting a precision medicine approach to enable targeted therapeutic intervention to improve sepsis outcomes.

Currently, progress in sepsis research is severely hampered by a heterogeneous disease phenotype. This heterogeneity manifests as a diversity of host responses that occur in different patients, making therapeutic management challenging (48, 49). Effective targeted therapy for sepsis requires an understanding of the heterogeneity of the individual host response (50). A precise approach using biomarkers allows for the identification of subgroups of patients with different host responses and specific individuals who are likely to benefit from a personalized therapy (51–53). Our genetic approach may classify individuals into subpopulations that differ in their susceptibility to sepsis, in the pathophysiology of sepsis, and in their response to a specific treatment according to their genomic information. Thus, our study not only provides insights into the pathogenesis of sepsis but also suggests a conceptual framework for the treatment of sepsis through a tailored approach.

## Materials and Methods

### Generation of Transgenic Mice.

Transgenic mice carrying HNP1–3-encoding genes *DEFA1/DEFA3* were generated using the P1 artificial chromosome construct (clone P1 34) (54). The transgenic construct was verified by sequencing and introduced into C57BL/6 mice. Transgene integration was identified by genomic PCR and high-throughput sequencing analysis. Mouse experiments were carried out in compliance with relevant animal-use guidelines and ethical regulations, and protocols for mouse experiments were approved by the institutional review boards of Zhejiang University School of Medicine, Hangzhou, China.

### Characterization of Transgenic Mice.

The genomic copy number of *DEFA1/DEFA3* was genotyped by means of quantitative real-time PCR, as described previously (27). The expression of the protein was determined in myeloid marrow precursors and peripheral blood polymorphonuclear cells, as well as in perfused lung, liver, and kidney by an immunostaining or immunoblotting test, as previously described (55).

### CLP-Induced Sepsis Model and Treatment Regimen.

Eight- to 10-wk-old transgenic mice or WT mice were subjected to CLP, as previously described (31). Mortality was assessed every 6–8 h. See *SI Appendix* for more details.

In some experiments for sepsis treatment, 300 μg of the B6B4 antibody was consecutively intravenously administered to mice via the tail vein immediately or at 12 h after CLP performance and then at 24 and 48 h after the first administration. As a control, equal amounts of normal saline or mouse IgG (I5381; Sigma-Aldrich) were administered in the same manner.

### Generation of BM Chimeric Mice.

To generate BM-chimeric mice, 8- to 10-wk-old recipient mice were lethally irradiated (8 Gy) using the RS 2000 X-ray Biological Irradiator (Rad Source Technologies) and reconstituted with three million bone marrow cells from donors by intravenous injection. Prophylactic antibiotics were administered during 1 wk before and the initial 2 wk after transplantation. Chimeras were used for further experiments 6–8 wk after the initial reconstitution.

### Histopathology.

For histopathology analysis, the lung, liver, and kidney tissues were fixed in a 4% paraformaldehyde-buffered solution for 24 h, embedded in paraffin and cut into 4-μm-thick sections, followed by H&E staining. For details, see *SI Appendix*.

### In Vivo Two-Photon Laser-Scanning Microscopy Imaging.

The dynamic changes in microvascular blood flow and leukocyte adhesion were captured by two-photon laser-scanning microscopy imaging in vivo (56). After they were anesthetized, the mice were placed on a heated stage and their core temperature was measured and maintained at 37 °C. A 1-cm incision was then made in the abdomen, and the mesentery was carefully externalized with a swab. The mesentery was covered with 1.5% (wt/vol) low-melting-point agarose saline, and glass (Φ = 6 mm) was placed horizontally on the agarose. In vivo imaging was performed using an upright Olympus two-photon laser scanning confocal microscope (BX61W1-FV1000; Olympus) with an excitation source of a Spectra-Physics MaiTai HP Deep See femtosecond Ti:Sa laser. The wavelength of the laser was set at 800 nm. A long-working distance (2 mm) water-immersion objective (25×, NA = 1.05) was adopted for in vivo bioimaging. For studies of leukocyte adhesion, leukocytes were labeled in vivo with an intravenous bolus injection of 100 µL of a 0.02% solution of rhodamine 6G (Sigma-Aldrich). The images were taken at scanning speed of 2 μs per pixel with a resolution of 1,024 × 1,024 pixels and 50 pictures in total. In vivo leukocyte adhesion and rolling velocity was measured manually using ImageJ (NIH) and Imaris (Olympus) software.

### In Vivo Permeability Assay.

The changes in vascular permeability were assessed with Evans blue dye and FITC-dextran leakage from the blood into tissues in vivo. For details, see *SI Appendix*.

### Flow Cytometry Analysis of Cell Pyroptosis.

For pyroptosis analysis, the single-cell suspension from mouse lung tissue was incubated with allophycocyanin-conjugated anti-CD31 antibody (551262; BD Biosciences) for 30 min. After washing, the cells were incubated with carboxyfluorescein FLICA (ImmunoChemistry Technologies), which binds to activated caspase-1, and PI, as described in the manufacturer’s instructions. The cells were then analyzed by flow cytometry. Acquisition was performed on 30,000 events using a BD LSR Fortessa (BD Biosciences), and the data were analyzed by BD FACSDiva (BD Biosciences). The percentage of both FLICA and PI double-stained events in CD31^+^ cells was taken as the percentage of pyroptotic endothelial cells.

### Modeling the Interaction Between HNP-1 and P2X7.

The sequences of hP2X7 (GenBank accession no. CAA73360.1) and mP2X7 (GenBank accession no. CAA08853.1) were obtained from the GenBank database. The 3D structures of hP2X7 and mP2X7 were built from the X-ray structure of the human P2X3 using the SWISS-MODEL web server (57). The crystal structure of the human HNP-1 was retrieved from the Protein Data Bank (PDB ID code 3GNY). The binding structure of HNP-1 in complex with hP2X7 or mP2X7 was predicted using the ZDOCK protein–protein docking web server (41). The most stable structures of HNP-1/hP2X7 and HNP-1/mP2X7 generated by ZDOCK were preoriented coordinates with respect to the normal membrane (*z* axis) from the orientations of proteins in the membranes database (58). Each of the oriented systems was inserted into a preequilibrated 1,2-dioleoyl-sn-glycero-3-phosphocholine bilayer surrounded by a 150-mM KCl aqueous salt solution using the Membrane Builder module in the CHARMM-GUI web server (59). The molecular mechanics parameters from the ff12SB force field, lipid14 force field, and Joung/Cheatham ion parameters for TIP3P water (ionsjc_tip3p) were assigned to the proteins, lipids, and ions, respectively, using the *LEaP* module of AMBER 16. The initial system sizes were ∼119 × 119 × 165 Å with more than 260,000 atoms.

MD simulations were carried out by using the *pmemd.cuda* module in AMBER 16. A prior multistage molecular mechanics minimization was utilized to remove unfavorable contacts. Afterward, each system was heated to 300 K over a period of 400 ps and equilibrated over 4 ns in the NPT ensemble (T = 300 K and P = 1 bar). Finally, each system was submitted to 90 ns NPT (T = 300 K and P = 1 bar) MD simulations. The binding free energy (Δ*G*_bind_) between HNP-1 and hP2X7 or mP2X7 was calculated by the MM/GBSA approach based on 200 snapshots that were evenly extracted from the last 20-ns MD trajectories, as described previously (60, 61).

### Statistical Analysis.

Data are presented as mean ± SEM unless indicated otherwise. Comparisons between two groups were performed by unpaired two-tailed *t* test. Comparisons between more than two groups were performed by one-way ANOVA with Bonferroni posttests, whereas comparisons with two or more independent variable factors were performed by two-way ANOVA followed by Bonferroni posttests. Survival data were analyzed with the log-rank test. Data were analyzed and plotted in Prism 7.0 software (GraphPad). *P* < 0.05 was considered statistically significant.

## Supplementary Material

Supplementary File

Supplementary File

Supplementary File

Supplementary File

Supplementary File

Supplementary File

Supplementary File

Supplementary File
