# Supplementary material for: Increased gene copy number of DEFA1/DEFA3 worsens sepsis by inducing endothelial pyroptosis
Source: Proc Natl Acad Sci U S A. 2019 Feb 4;116(8):3161–70. doi: 10.1073/pnas.1812947116 (PMC6386704; doi:10.1073/pnas.1812947116)
Supplement: Supplementary File [file pnas.1812947116.sapp.pdf]

PNAS
www.pnas.org

Increased gene copy number of *DEFA1/DEFA3* worsens sepsis through inducing endothelial pyroptosis

QiXing Chen[a], Yang Yang[b], JinChao Hou[b], Qiang Shu[a], YiXuan Yin[c], Weitao Fu[c], Feng Han[c], TingJun Hou[c,d], CongLi Zeng[b], Elizabeta Nemeth[e], Rose Linzmeier[e], Tomas Ganz[e,1], XiangMing Fang[a,b,1]

[a]Department of Clinical Research Center, Children's Hospital, Zhejiang University School of Medicine, Hangzhou 310052, China; [b]Department of Anesthesiology, The First Affiliated Hospital, Zhejiang University School of Medicine, Hangzhou 310003, China; [c]College of Pharmaceutical Sciences, Zhejiang University, Hangzhou 310058, China; [d]Institute of Functional Nano and Soft Materials, Soochow University, Suzhou 215123, China; [e]Department of Medicine, David Geffen School of Medicine at UCLA, Los Angeles, CA 90095, USA.

[1]Correspondence: XiangMing Fang, M.D., Department of Anesthesiology, The First Affiliated Hospital, School of Medicine, Zhejiang University, QingChun Road 79, 310003 Hangzhou, China. Phone: 0086-571-88208021; E-mail: xmfang@zju.edu.cn; Tomas Ganz, M.D., Department of Medicine, David Geffen School of Medicine at UCLA, Los Angeles, CA 90095, USA. Phone: 310-825-7499; E-mail: TGanz@mednet.ucla.edu.

This PDF file includes:

- Supplementary text
- Figs. S1 to S16
- Captions for movies S1 to S7
- References for SI reference citations

Other supplementary materials for this manuscript include the following:

- Movies S1 to S7

www.pnas.org/cgi/doi/10.1073/pnas.1812947116

Supplementary Information Text

Materials and Methods

Mice. *P2x7*$^{-/-}$ mice and *Nlrp3*$^{-/-}$ mice have been described previously (1, 2). *Casp11*$^{-/-}$ mice were a gift from F. Shao (National Institute of Biological Sciences, Beijing, China). Mouse genotypes were assessed by PCR. WT mice on a C57BL/6 genetic background were purchased from Shanghai SLAC Animal Laboratory (Shanghai, China).

CLP-induced sepsis model. To perform CLP, the cecum was exposed through a 1.5-cm longitudinal incision in the lower quadrants of the abdomen. The cecum was ligated with 4-0 silk suture and punctured doubly with a 22-gauge needle. After puncturing, the cecum was gently squeezed to ensure the patency of holes by observing the extrusion of feces. The cecum was then replaced in the peritoneal cavity, and the incision was closed with surgical suture. After surgery, 1 ml of pre-warmed saline per 20 g body weight was subcutaneously administered. Sham-treated mice underwent the same procedures but without ligation and puncture of the cecum.

***Escherichia coli* peritonitis model.** For the bacterial peritonitis model, eight to ten-week-old transgenic mice or WT mice were injected intraperitoneally with a suspension of live *E. coli* (ATCC 25922; 4×10^6 colony forming units (CFU) per mouse). For growth of *E. coli*, a single colony from an agar plate was subcultured in pre-warmed Luria-Bertani (LB) medium and incubated to mid-exponential growth phase in a 37 °C shaking incubator. The cultures were then centrifuged at 4000 rpm for 10 min, and the pellets were washed with phosphate-buffered saline (PBS). The bacterial concentration was estimated photometrically, and working dilutions were prepared in 200 μl of PBS.

Histopathology. For histopathology analysis, the lung, liver and kidney tissues were fixed in a 4% paraformaldehyde buffered solution for 24 h and then processed using standard procedures. Tissues were embedded in paraffin and cut into 4-μm-thick sections, followed by hematoxylin and eosin (H&E) staining. The kidney tissue sections were also subjected to Periodic acid-Schiff staining. Histological examination was quantitatively scored in a random order. We assessed and scored histological lung injury on a 5-point scale: 0, minimal damage; 1 to > 2, mild damage; 2 to > 3, moderate damage; 3 to > 4, severe damage; and 4 +, maximal damage (3). Liver injury was assessed for necrosis by standard morphologic criteria (loss of architecture, vacuolization, karyolysis, increased eosinophilia), and the extent of necrosis was estimated by assigning a severity score on a scale from 0 - 4 as previously described: 0 = absent, 1 = mild, 2 = moderate, 3 = severe, and 4 = total necrotic destruction of the liver (4). Kidney injury was evaluated by using the acute tubular necrosis score according to any of the following: proximal tubule dilation, brush-border damage, proteinaceous casts, interstitial widening, and necrosis (0, no injury; 1, less than 10%; 2, 10 to 25%; 3, 26 to 45%; 4, 46% to 75%; 5, > 75%) (5, 6). All scoring was based on ten randomly selected images under high-power field microscopy for each sample. Semi-quantitative morphometric examinations were performed in a blinded manner and without knowledge of the group identity or experimental model.

Bacterial count determination. To determine the bacterial counts, samples of peritoneal lavage fluid and blood were serially diluted 10-fold with sterile PBS. Log dilutions were plated on LB agar plates, which were incubated at 37 °C for 12 to 16 h under aerobic conditions. The numbers of bacterial colonies were calculated as CFU, and data were log transformed for statistical analysis.

Inflammatory mediator measurements. Plasma levels of inflammatory mediators were determined using a cytometric bead array assay (BD Biosciences) or using a commercially available enzyme-linked immunosorbent assay (ELISA) kits (R&D Systems) according to the manufacturer's instructions.

Macrophage and monocyte depletion by clodronate liposome injection. For *in vivo* macrophage and monocyte depletion, mice were injected intravenously with 200 μl of clodronate or PBS liposomes (FormuMax Scientific) via the tail vein 48 h before performance of CLP. Depletion efficiency was confirmed by flow cytometry analysis.

Immunohistochemistry. For immunohistochemistry staining, the tissue samples were sectioned in 4-μm slices and subjected to a standard immunohistochemical procedure. The leukocyte samples isolated from peripheral blood were spread evenly on slides. After fixed with 95% ethanol for 15 min, the samples were subjected to a standard immunohistochemical procedure. The expression of ICAM-1 and VCAM-1 in the tissues as well as LFA-1 and VLA-4 in the leukocytes was detected with monoclonal rat antibody (for ICAM-1; Abcam, ab25375), monoclonal rabbit antibody (for VCAM-1; Abcam, ab134047), monoclonal mouse antibody (for LFA-1; Abcam, ab13219) and polyclonal rabbit antibody (for VLA-4; Abcam, ab202969) respectively, and related biotinylated second antibodies (ZSGB-BIO). After incubation with diaminobenzidine as the chromatogen followed by hematoxylin counterstaining, samples were visualized using an Olympus microscope (Olympus). At least five images per sample were randomly photographed using a camera and a microscope-attached modular photomicrographic system. The intensity of staining was then quantified using Image-Pro Plus 6.0 analysis system (Media Cybernetics Inc.). The average optical density per section was calculated by dividing the sum integrated optical density by the sum area.

Immunofluorescence. For immunofluorescence staining of tissues, the paraffin-embedded tissues were deparaffinized, rehydrated, and subjected to antigen retrieval by heating for 10 min in citrate buffer in a microwave oven. After blocking with 10% bovine serum for 1 h at room temperature, the sections were incubated with primary antibody against mouse CD31 (Abcam, ab28364) or P2X7 (Alomone Labs, APR-004) at 4 °C overnight, followed by exposure to FITC- or Cy3-conjugated secondary antibodies (Jackson ImmunoResearch). Nuclear staining was carried out with 4',6-diamidino-2-phenylindole (Invitrogen) using Prolong Gold mounting medium (Invitrogen). All images were collected with a Nikon A1 confocal microscope (Nikon), and the fluorescence intensity was analyzed using Image J software (NIH). The fluorescence signals in at least five different high-power fields from each sample were quantified. Image analysis was performed by the investigators, who were blinded to the genotype of the animals and the identity of the samples.

***In vivo* permeability assay.** The changes in vascular permeability were assessed with Evans blue dye (EBD) and fluorescein isothiocyanate (FITC)-dextran leakage from the blood into tissues *in vivo*. At 72 h after CLP, mice were anesthetized, and EBD (20 mg/kg; Sigma-Aldrich) in a volume of 100 μl was administered intravenously by tail vein injection. Thirty minutes after dye injection, the mice were transcardially perfused with normal saline through the left ventricle until blood was completely eliminated. Tissues of lung, kidney and mesentery were harvested and dried at 55 °C. Twenty-four hours later, the tissues were weighed and homogenized in 1 ml of formamide (Sigma-Aldrich) and then incubated at 55 ºC for 48 h. Supernatants were collected after centrifugation at 5000 *g* for 30 min. The EBD in the supernatant was quantitated using a dual wavelength (620 and 740 nm) spectrophotometric method, correcting for contaminating heme pigments by using the formula OD_{620} (EBD) = $OD_{620} - (1.426 \times OD_{740} + 0.030)$ (7). The extravasated EBD concentration in the tissue homogenate was calculated from a standard curve of EBD and expressed as the dye incorporated per mg of tissue. Alternatively, 200 μl of FITC-conjugated dextran (40 kDa; 5 mg/ml; Sigma-Aldrich) was intravenously injected via the tail vein 30 min before sacrifice, and then the mice were transcardially perfused. The tissue was harvested and homogenized, and the fluorescence intensity of FITC-dextran in the tissue homogenate was measured using a fluorometer (Multi-Mode Microplate Reader SynergyMax M5; Molecular Devices) with 488 nm (excitation) and 520 nm (emission) filters.

Dissociation of lung cells. After the mice were anesthetized and the chest cavity opened, the lung was removed and rinsed with Hank's Balanced Salt Solution. The sample was then digested using the Mouse Lung Dissociation Kit (Miltenyi Biotec) on the gentleMACS dissociator (Miltenyi Biotec). The tissue was incubated in the enzyme mixture for 30 min at 37 °C after homogenizing with the gentleMACS program "m_lung_01". The gentleMACS program "m_lung_02" was applied for final homogenization. After termination of the program, the cell suspension was passed through a MACS SmartStrainer (70 μm), and the cells were processed immediately for further applications.

Flow cytometry analysis of cell apoptosis. For apoptosis analysis, single-cell suspensions from mouse lung tissue were incubated with allophycocyanin-conjugated anti-CD31 antibody for 30 min. After washing, the cells were incubated with FITC-conjugated Annexin V/PI from the Apoptosis Detection Kit II (BD Biosciences) following the manufacturer's instruction. The cells were then analyzed by flow cytometry. Acquisition was performed on 30,000 events using a BD LSR Fortessa, and the data were analyzed by BD FACSDiva. The percentage of both Annexin V and PI double-stained events among the $CD31^+$ cells was taken as the percentage of apoptotic endothelial cells.

Cell culture and treatment. Murine lung microvascular endothelial cells (MLMECs; BeNa Culture Collection) were maintained in Dulbecco's Modified Eagle's Medium supplemented with 10% FBS and cultured in a humidified incubator with 5% CO_2 at 37 °C. On the day before stimulation, the cells were seeded in 12-well plates (2×10^5 cells/well) for an

overnight incubation and then stimulated with 1 μg/ml LPS and 100 ng/ml IFN-γ for 48 h. The medium supernatants were subsequently removed, and the cells were rinsed twice with pure medium. At this point, HNP-1 was added to the pure fresh medium, and the cells were incubated for another 24 h. The cells and supernatant were then harvested for further analysis.
For *P2x7* knockdown experiments, MLMECs were plated in 12-well plates to 80% confluence, and then confluent cells were transfected with either 10 pM of mouse *P2x7* siRNA (Santa Cruz biotechnology) or control siRNA (Santa Cruz Biotechnology) using Lipofectamine 2000 (Life Technology) according to the manufacturer's instructions.
For antibody blocking, HNP-1 was pre-incubated with mouse IgG (Sigma-Aldrich, I5381), or antibody B6B4 or A4B11 (1:10 mass ratio) at 37 °C for 2 h, and then the mixture was added to the cells for treatment.

Immunofluorescence. For immunofluorescence staining of P2X7 in cultured MLMECs, the cells were grown in a 12-well plate (1×10^5 cells/well) with or without 1 μg/ml LPS and 100 ng/ml IFN-γ stimulation at 37 °C for 48 h. The cells on the slide were fixed with 4% formaldehyde at room temperature for 15 min, permeabilized with 0.5% Triton X-100 for 10 min, blocked with 10% bovine serum albumin at room temperature for 45 min, and incubated with primary antibody against P2X7 at 4 °C overnight. The cells were then incubated with Cy3-conjugated secondary antibody (Jackson ImmunoResearch) at 37 °C for 1 h. Nuclei were stained with 4',6-diamidino-2-phenylindole (Invitrogen). All images were collected with a Nikon A1 confocal microscope (Nikon), and the fluorescence intensity was analyzed using Image J software (NIH). The fluorescence signals in at least five different high-power fields from each sample were quantified.

Western blot analysis. For detection of cellular levels of activated caspase-1, the treated cells were lysed in RIPA Lysis and Extraction Buffer (Beyotime) supplemented with 100 mg/ml phenylmethylsulfonyl fluoride. The protein concentration was determined using a BCA Protein Assay kit (Pierce Biotechnology). After denaturation, 40 μg of total protein was fractionated on a 12% NuPage Tris-Bis gel and then transferred to a polyvinylidene difluoride membrane. After blocking with 5% non-fat dry milk at room temperature for 2 h, the membranes were incubated with anti-caspase-1 antibody (Abcam, ab179515), anti-caspase-11 antibody (Sigma-Aldrich, C1354), anti-caspase-3 antibody (Epitomics, 1476-1), anti-gasdermin D (GSDMD) antibody (Sigma-Aldrich, G7422), anti-tubulin antibody (Abcam, ab52866), or anti-actin antibody (Sigma-Aldrich, A5316) at 4 °C overnight, followed by another 2-h incubation with the related secondary antibody at room temperature. The blots were developed using the ECL Chemiluminescence detection kit (Biological Industries) and visualized using X-ray film. α-tubulin or β-actin was used as a loading control.
Cellular protein levels of HNPs were detected as previously described (8). Briefly, bone marrow cells were isolated and prepared from femurs of young adult transgenic mice and WT mice. Mature myeloid cells were separated by laying cells on a Percoll/PBS solution and centrifugation at 1000 *g* for 20 minutes. The total number of mature myeloid cells was determined using a hemocytometer. The cells were centrifuged at 1000 g for 5 min at 4°C and the supernatant was removed. The number of cells was then adjusted to 5×10^5 cells per tube with 1% acetic acid. HNPs were extracted from the cells placed on ice by sonication, using 3

pulses lasting 10 seconds each with a Sonic Dismembrator. Protein was extracted overnight at 4°C on a rotator (Eppendorf). After centrifuging at 14000 rpm for 10 min at 4°C, the supernatant was carefully removed into a fresh tube. The protein concentration was determined using a BCA Protein Assay kit (Pierce Biotechnology). After denaturation in the appropriate gel loading solution, 60 μg of total protein was separated on a 10-20% Tricine Protein Gels (Life Technology) and then transferred to a polyvinylidene difluoride membrane. Western blot was then performed as described above using anti-HNP1 antibody B6B4 (described below).

Generation and purification of monoclonal antibodies. BALB/c mice (6 - 8 weeks old) were immunized with the synthetic peptide antigen (100 μg/injection) conjugated to Keyhole limpet hemocyanin (Sigma-Aldrich). The peptides were mixed with Freund's complete adjuvant (Sigma-Aldrich) in the initial immunization and with Freund's incomplete adjuvant (Sigma-Aldrich) in the remaining immunizations. Twenty-one days later, the mice were again immunized with 50 μg conjugated antigen, followed by two additional injections every two weeks. Blood was collected from mouse tails on day 7 after immunization, and serum anti-HNP-1-specific antibodies were determined by indirect ELISA. Splenocytes from the mice with the highest antibody titers specific for the synthetic peptide were fused with Sp2/0 myeloma cells (American Type Culture Collection) at a 10:1 ratio using polyethylene glycol 1500. Supernatants from individual hybridomas were screened by indirect ELISA for their reactivity with the synthetic peptide. Hybridoma pools showing a positive signal were selected for clonal selection through limiting dilution. Finally, approximately 13 monoclonal hybridoma lines were established. Among them, two lines that showed high affinity to the synthetic peptide were selected. The selected clones were then injected intraperitoneally into BALB/c mice to produce ascitic fluid. The monoclonal antibodies were purified from the ascites using a protein G-Sepharose column (Pierce) according to the manufacturer's instructions.

Bio-Layer Interferometry. Interactions between HNP-1 and the monoclonal antibodies were studied by Bio-Layer Interferometry on an Octet K2 system (ForteBio). The assay was performed in black 96-well plates (Greiner Bio-One) at 37 °C. The total working volume for the samples or buffer was 0.21 ml per well, and the rpm setting for each equilibrium, loading, association and dissociation step was 1000 rpm. Prior to each assay, the Octet aminopropyl silane biosensor (ForteBio) was pre-wetted in PBS for at least 10 min, followed by equilibration with PBS for 30 s. Kinetic assays were performed by first capturing antibody using Octet aminopropyl silane biosensors, followed by a baseline step of 240 s in PBS containing 0.02% Tween-20. Subsequently, the association of the antibody with HNP-1 in a concentration range of 500 nM, 250 nM, 125 nM, 62.5 nM and 31.25 nM was performed for 150 s. Finally, dissociation was monitored with PBS containing 0.02% Tween-20 for 100 s. The antibody-captured sensors were also dipped in wells containing PBS with 0.02% Tween-20 to allow for single reference subtraction to compensate for the natural dissociation of captured antibody. All measurements were performed in triplicate. Results were analyzed using ForteBio Octet data analysis 7.0 software.

Supplementary Information Figures

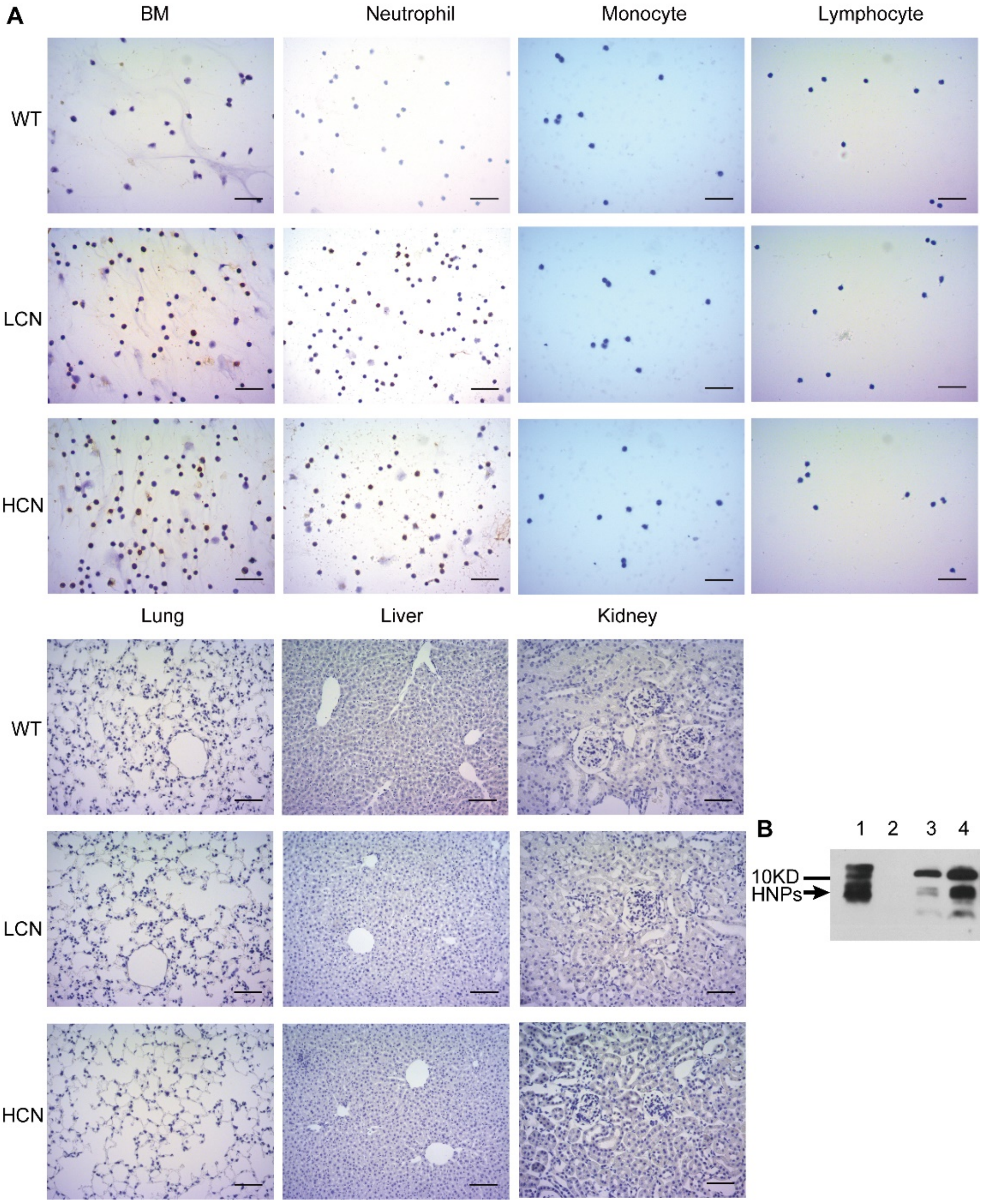

Figure S1. HNP1-3 is present in two founder transgenic mice, mice with a low copy number (LCN) of *DEFA1/DEFA3* and mice with a high copy number (HCN) of *DEFA1/DEFA3*. (*A*) The expression of HNP1-3 in transgenic mice and wild-type (WT) mice was detected using immunohistochemistry analysis in bone marrow cells, peripheral blood neutrophils, peripheral blood macrophages, peripheral blood lymphocytes, and perfused lung, liver and kidney tissues. The primary antibody used is from Santa Cruz Biotechnology (SC-22915). Data are representative of two independent experiments. Scale bars, 20 μm. (*B*) HNPs were detected in extracts from neutrophils isolated from bone marrow cells from WT mice (lane 2), mice with LCN of *DEFA1/DEFA3* (lane 3) and mice with HCN of *DEFA1/DEFA3* (lane 4) followed by Western blot using anti-HNP1 monoclonal antibody B6B4 described in present study. Protein extract from neutrophils of human blood donor was prepared as a control (lane 1).

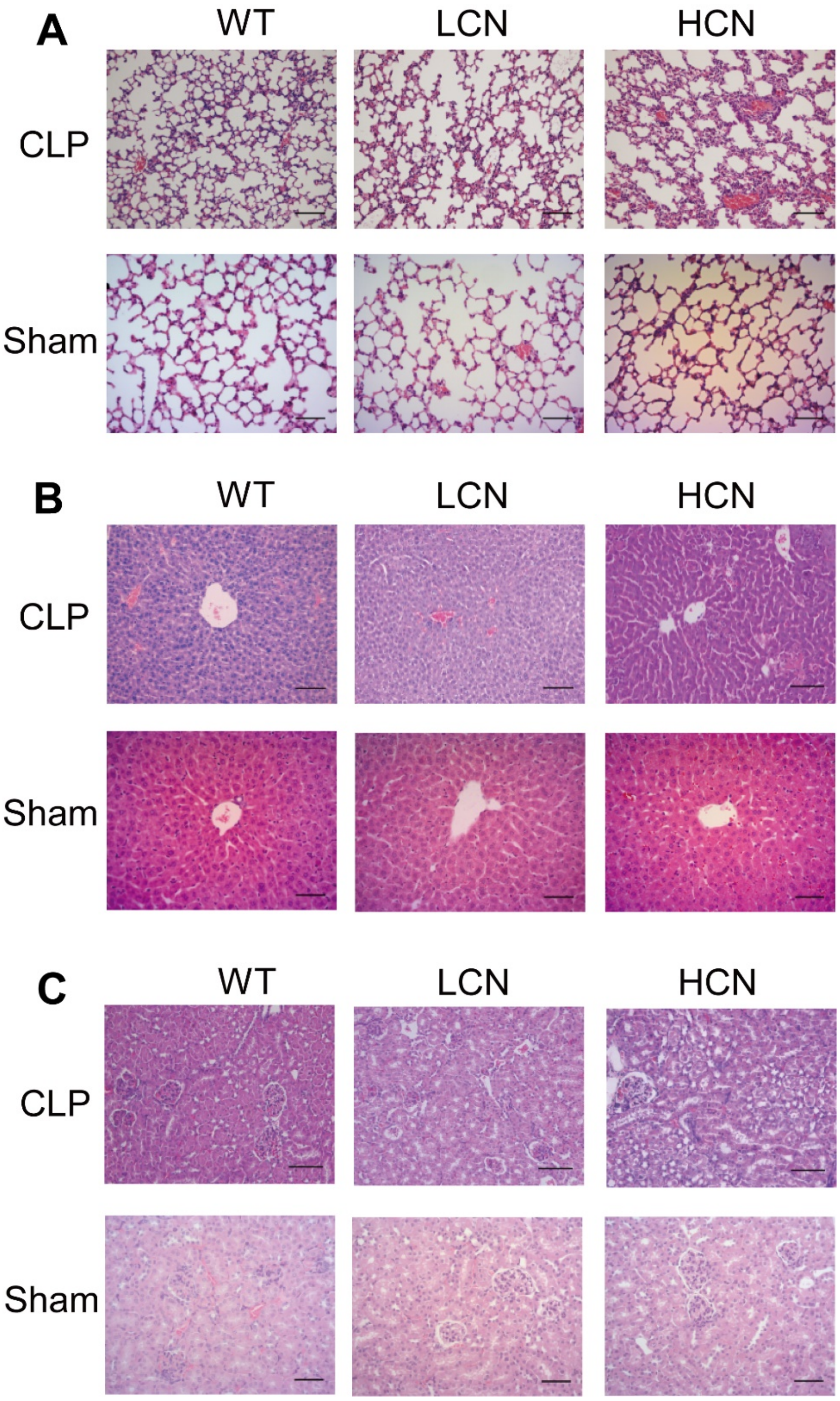

Figure S2. Increased gene copy number of *DEFA1/DEFA3* aggravates vital organ damage during sepsis. (*A*) Representative images of hematoxylin and eosin (HE)-stained lung sections of WT mice, mice with LCN of *DEFA1/DEFA3*, and mice with HCN of *DEFA1/DEFA3* at 72 h after CLP or sham operation. Scale bars, 20 μm. (*B*) Representative images of HE-stained liver sections of WT mice, mice with LCN of *DEFA1/DEFA3*, and mice with HCN of *DEFA1/DEFA3* at 72 h after CLP or sham operation. Scale bars, 20 μm. (*C*) Representative images of HE-stained kidney sections of WT mice, mice with LCN of *DEFA1/DEFA3*, and mice with HCN of *DEFA1/DEFA3* at 72 h after CLP or sham operation. Scale bars, 20 μm.

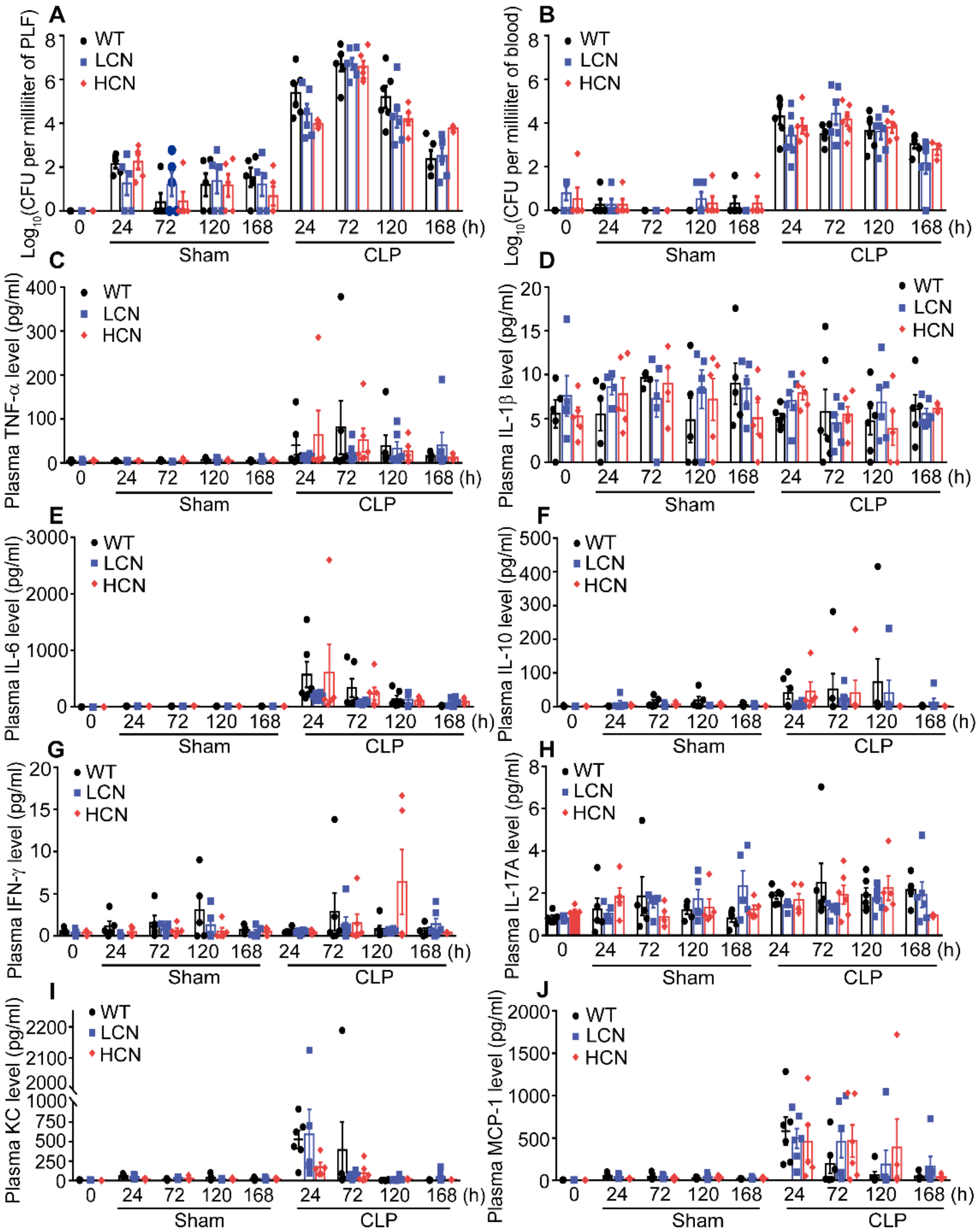

Figure S3. Pathophysiological characterization of *DEFA1/DEFA3* transgenic mice after sepsis. (*A* and *B*) Bacterial load in peritoneal lavage fluid (PLF) (*A*) and peripheral blood (*B*) over time after performance of CLP or sham operation in WT mice, mice with LCN of *DEFA1/DEFA3*, and mice with HCN of *DEFA1/DEFA3*. (*C-J*) Plasma levels of inflammatory mediators TNF-α (*C*), IL-1β (*D*), IL-6 (*E*), IL-10 (*F*), IFN-γ (G) and IL-17A (H), as well as KC (I) and MCP-1 (J) over time after performance of CLP or sham operation in WT mice, mice with LCN of *DEFA1/DEFA3*, and mice with HCN of *DEFA1/DEFA3*. In *A-F*, *n* = 5 for each group at 0 h, *n* = 5 for each sham group at each time point, *n* = 6 for WT mice and mice carrying LCN of *DEFA1/DEFA3* at 24 h, 72 h and 120 h after CLP, and for mice carrying LCN of *DEFA1/DEFA3* at 168 h after CLP and for mice carrying HCN of *DEFA1/DEFA3* at 72 h, *n* = 5 for mice carrying HCN of *DEFA1/DEFA3* at 24 h, 120 h and WT mice at 168 h after CLP, *n* = 3 for mice carrying HCN of *DEFA1/DEFA3* at 168 h after CLP. In *A-F*, data are shown as the mean ± s.e.m. using bar graphs overlaying the corresponding dot plots.

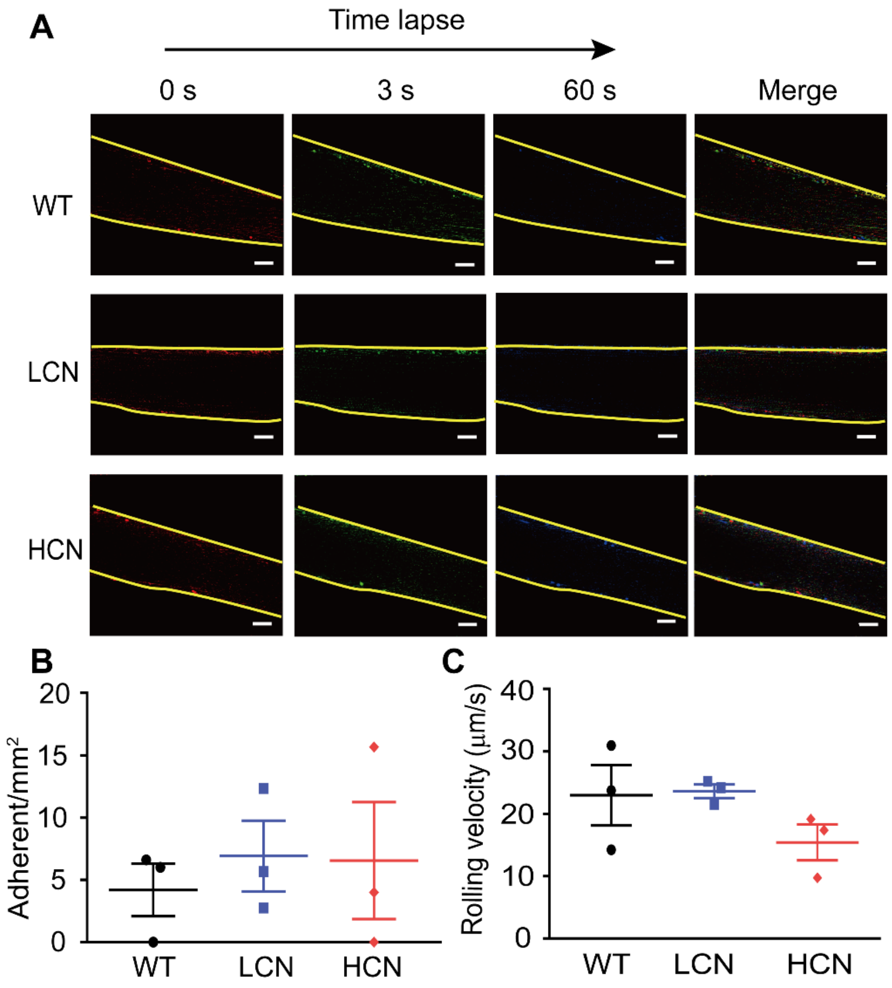

Figure S4. Leukocyte adhesion in transgenic mice and WT mice at 72 h following sham operation. (*A*) Representative *in vivo* time-lapse imaging with two-photon laser scanning microscopy showing leukocyte adhesion in mesenteric vessels in WT mice, mice with LCN of *DEFA1/DEFA3*, and mice with HCN of *DEFA1/DEFA3* at 72 h after sham operation. Images are individual frames from a continuous time-lapse movie. Times are given in seconds. In each group, the first panel shows baseline adhesion in the vessel, the second and third panels show leukocyte adhesion at 3 s and 60 s after the baseline imaging, and the fourth panel shows the merged images. Red indicates leukocyte adhesion at baseline; green indicates leukocyte adhesion at 3 s after baseline; blue indicates leukocyte adhesion at 60 s after baseline; yellow dashed lines indicate the vessel. Scale bar, 50 μm. (*B*) Quantitative measurements of firmly adhering leukocytes in WT mice, mice with LCN of *DEFA1/DEFA3*, and mice with HCN of *DEFA1/DEFA3* at 72 h after sham operation. Firmly adherent leukocytes from at least three fields of view per mouse were counted as the number of cells per mm^2 of vascular surface area. (*C*) Quantitative measurements of leukocyte rolling velocities in WT mice, mice with LCN of *DEFA1/DEFA3*, and mice with HCN of *DEFA1/DEFA3* at 72 h after sham operation. In *B* and *C*, $n = 3$ for each sham group.

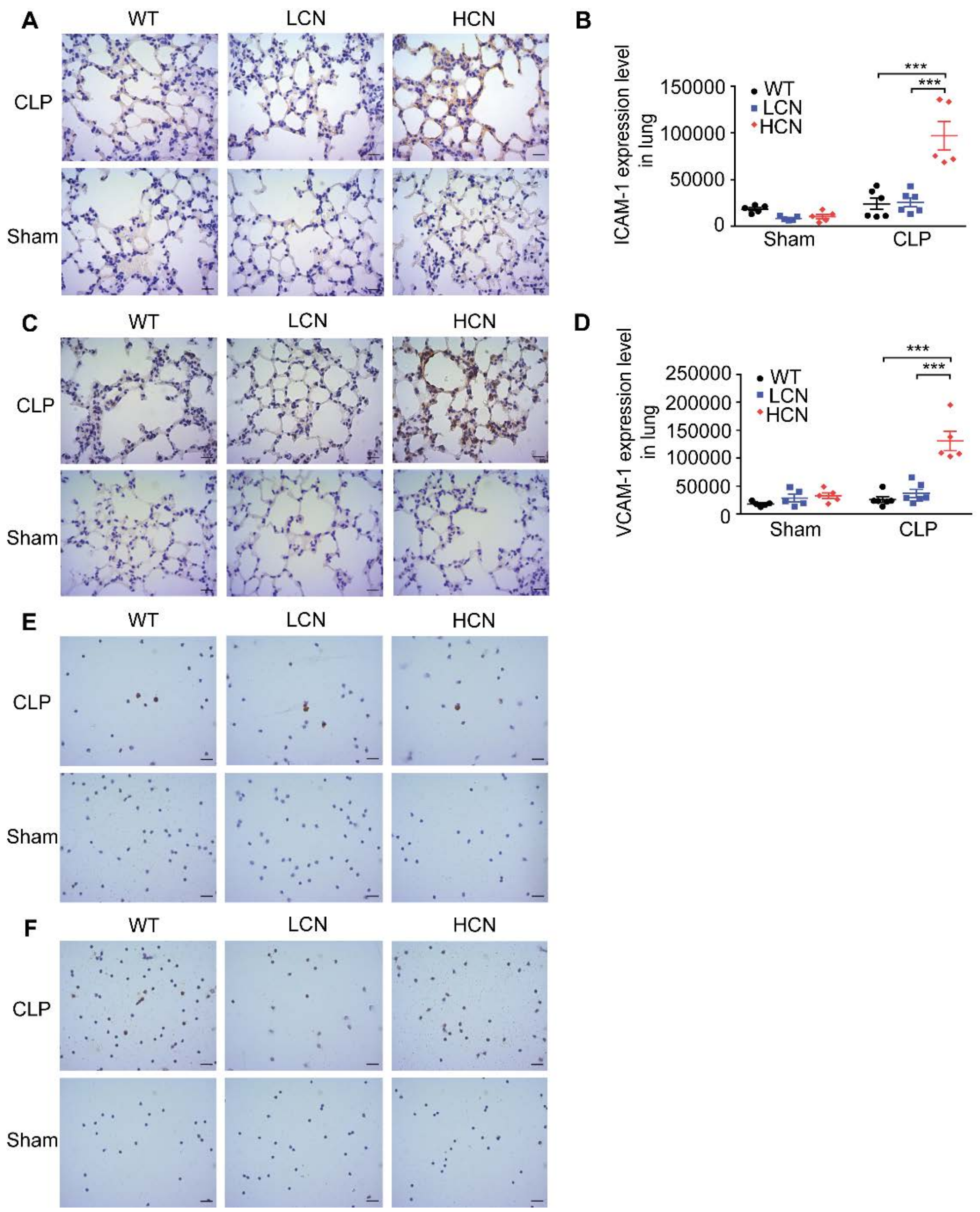

Figure S5. Expression of endothelial cell and leukocyte adhesion molecules in transgenic mice and WT mice at 24 h following CLP or sham operation. (*A* and *B*) Representative immunohistochemistry images of ICAM-1 expression in the lungs of WT mice, mice with LCN of *DEFA1/DEFA3*, and mice with HCN of *DEFA1/DEFA3* (*A*), and quantification of the expression level (*B*). Scale bars, 20 µm. . ***$P < 0.001$, two-way ANOVA with Bonferroni post tests. (*C* and *D*) Representative immunohistochemistry images of VCAM-1 expression in the lungs of WT mice, mice with LCN of *DEFA1/DEFA3*, and mice with HCN of *DEFA1/DEFA3* (*C*), and quantification of the expression level (*D*). Scale bars, 20 µm. . ***$P < 0.001$, two-way ANOVA with Bonferroni post tests. (*E* and *F*) Representative immunohistochemistry images of LFA-1 expression (*E*) and VLA-4 expression (*F*) in peripheral blood leukocytes of WT mice, mice with LCN of *DEFA1/DEFA3*, and mice with HCN of *DEFA1/DEFA3*. Scale bars, 20 µm. In *B* and *D*, data are presented as dot plots with horizontal bars representing means ± s.e.m.; $n = 5$ for each sham group and the CLP group of mice with HCN of *DEFA1/DEFA3*, $n = 6$ for each CLP and sham group. In *E* and *F*, $n = 3$ for each sham group, $n = 5$ for each CLP group.

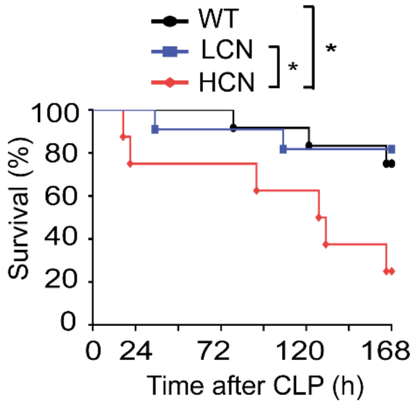

Figure S6. Depletion of monocyte/macrophage does not affect the outcome of transgenic mice during sepsis. Survival in WT mice (n = 12), mice with LCN of *DEFA1/DEFA3* (n = 11) and mice with HCN of *DEFA1/DEFA3* (n = 8) that were injected with clodronate liposomes at 48 h before and 48 h after performance of CLP. *P = 0.022 between WT mice and mice with HCN of *DEFA1/DEFA3*, *P = 0.016 between mice with LCN of *DEFA1/DEFA3* and mice with HCN of *DEFA1/DEFA3*, log-rank test.

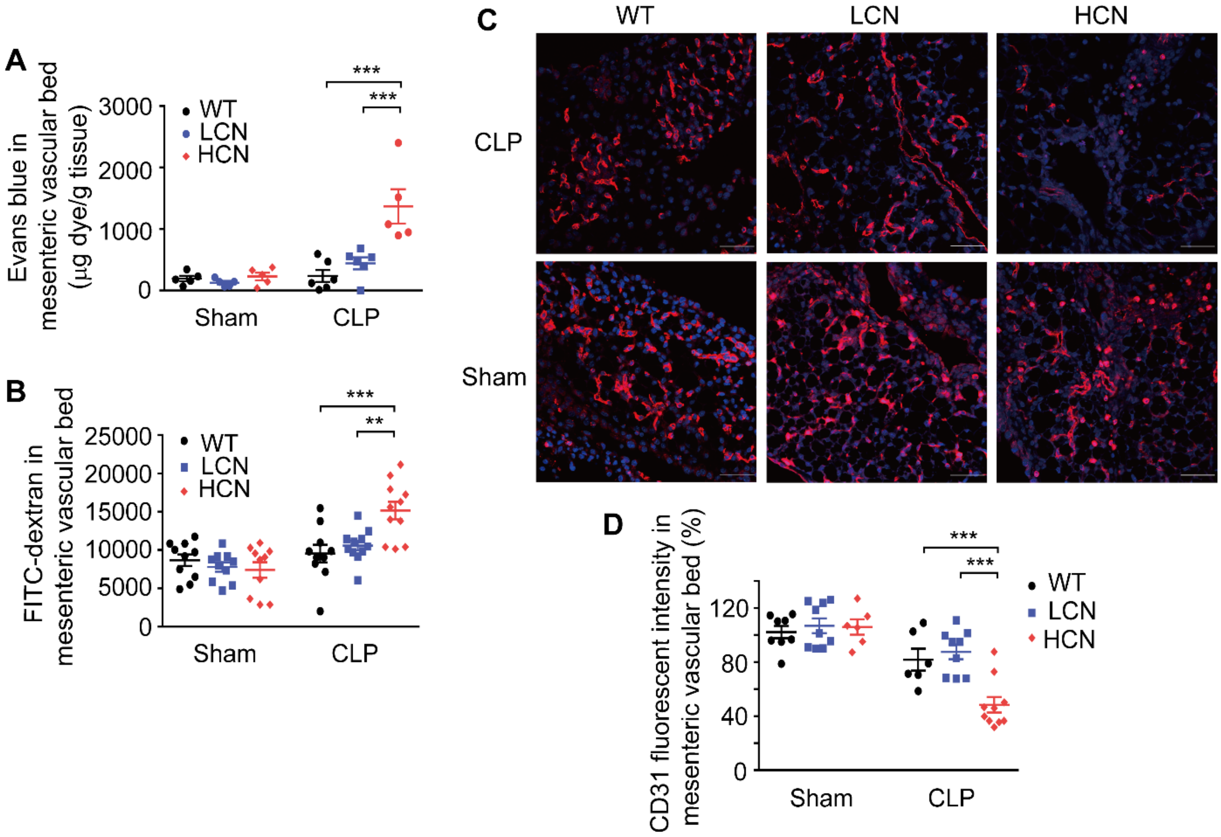

Figure S7. Increased gene copy number of *DEFA1/DEFA3* leads to mesenteric microvascular endothelial barrier dysfunction after sepsis. (*A*) *In vivo* Evans blue dye permeability assay at 72 h after performance of CLP or sham operation in WT mice (n = 6 for CLP, and n = 5 for sham), mice with LCN of *DEFA1/DEFA3* (n = 6 for CLP, and n = 5 for sham), and mice with HCN of *DEFA1/DEFA3* (n = 5 for each CLP and sham group). ***$P < 0.001$, two-way ANOVA with Bonferroni post tests. (*B*) *In vivo* fluorescein isothiocyanate (FITC)-dextran permeability assay at 72 h after performance of CLP or sham operation in WT mice (n = 10 for each CLP and sham group), mice with LCN of *DEFA1/DEFA3* (n = 11 for CLP, and n = 10 for sham), and mice with HCN of *DEFA1/DEFA3* (n = 11 for CLP, and n = 10 for sham). **$P = 0.0018$, ***$P < 0.001$, two-way ANOVA with Bonferroni post tests. (*C* and *D*) Representative images (*C*) and comparisons (*D*) of $CD31^+$ endothelial cells at 72 h after performance of CLP or sham operation in WT mice (n = 6 for CLP, and n = 8 for sham), mice with LCN of *DEFA1/DEFA3* (n = 9 for each CLP and sham group), and mice with HCN of *DEFA1/DEFA3* (n = 6 for CLP, and n = 10 for sham). $CD31^+$ endothelial cells were quantified by the fluorescence intensity in at least five different high-power fields from each slide. Red indicates CD31 staining; blue indicates DAPI staining. Scale bars, 50 µm. ***$P < 0.001$, two-way ANOVA with Bonferroni post tests. In *A*, *B* and *D*, data are presented as dot plots with horizontal bars representing means ± s.e.m.

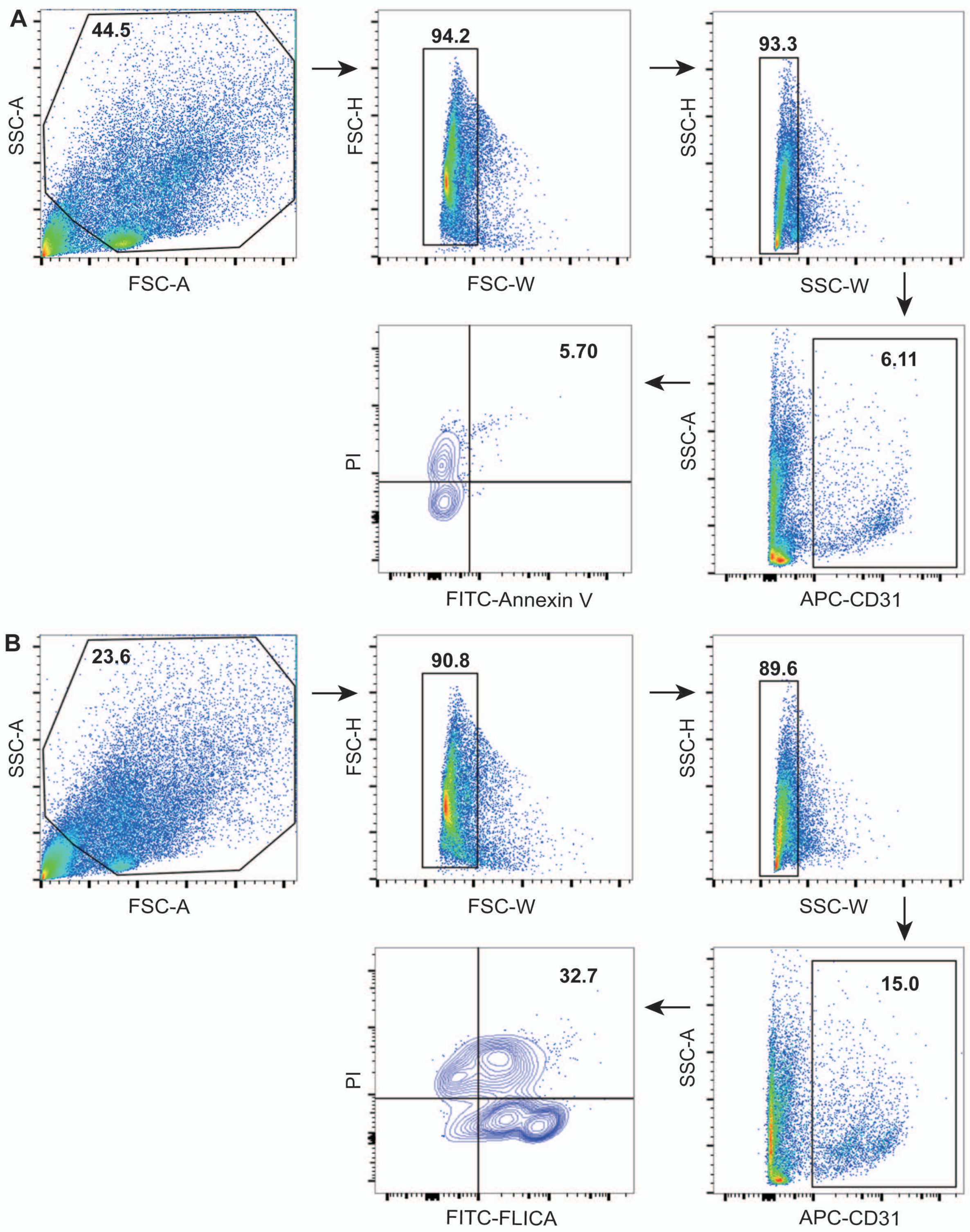

Figure S8. Flow cytometry gating strategy for identifying apoptotic and pyroptotic endothelial cells in murine lung. (*A*) Flow cytometry plot indicating the gating strategy to identify $CD31^+AnnexinV^+PI^+$ cells from murine lungs. The lungs were obtained from CLP-performed or sham-operated WT mice, mice with LCN of *DEFA1/DEFA3* and mice with HCN of *DEFA1/DEFA3*. (*B*) Flow cytometry plot indicating the gating strategy to identify $CD31^+FLICA^+PI^+$ cells from murine lungs. The lungs were obtained from CLP-performed or sham-operated WT mice, mice with LCN of *DEFA1/DEFA3* and mice with HCN of *DEFA1/DEFA3*.

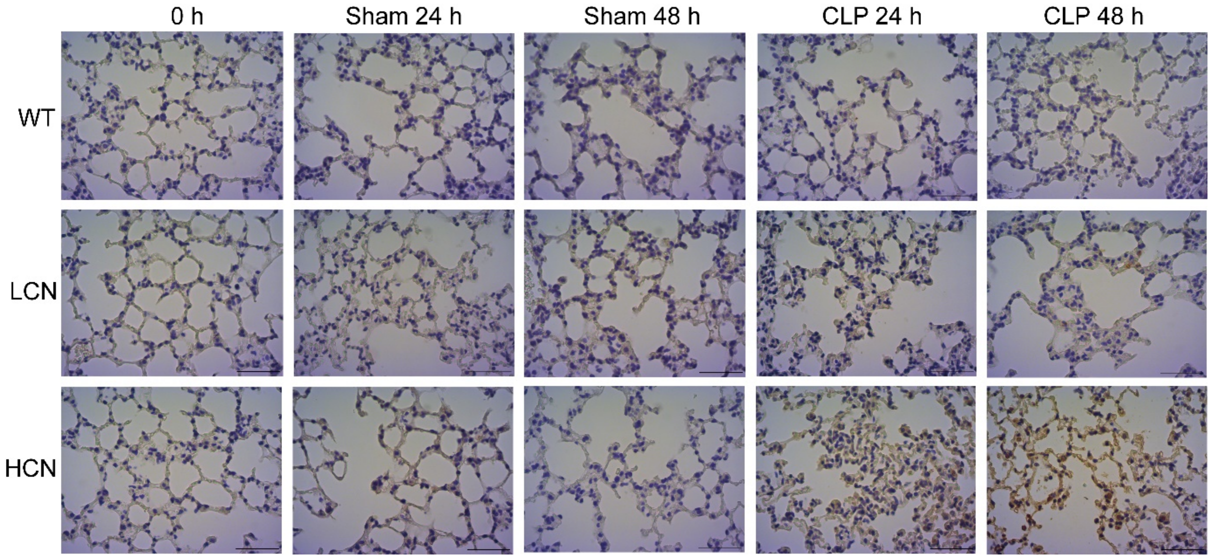

Figure S9. Expression of HNP1-3 in transgenic mice after sepsis. Representative immunohistochemistry images of HNP1-3 expression in the lungs of WT mice (*n* = 3 for each CLP and sham group), mice with LCN of *DEFA1/DEFA3* (*n* = 3 for each CLP and sham group), and mice with HCN of *DEFA1/DEFA3* (*n* = 3 for each CLP and sham group) at 0 h, and 24 h, 48 h after CLP or sham operation. Scale bars, 50 μm.

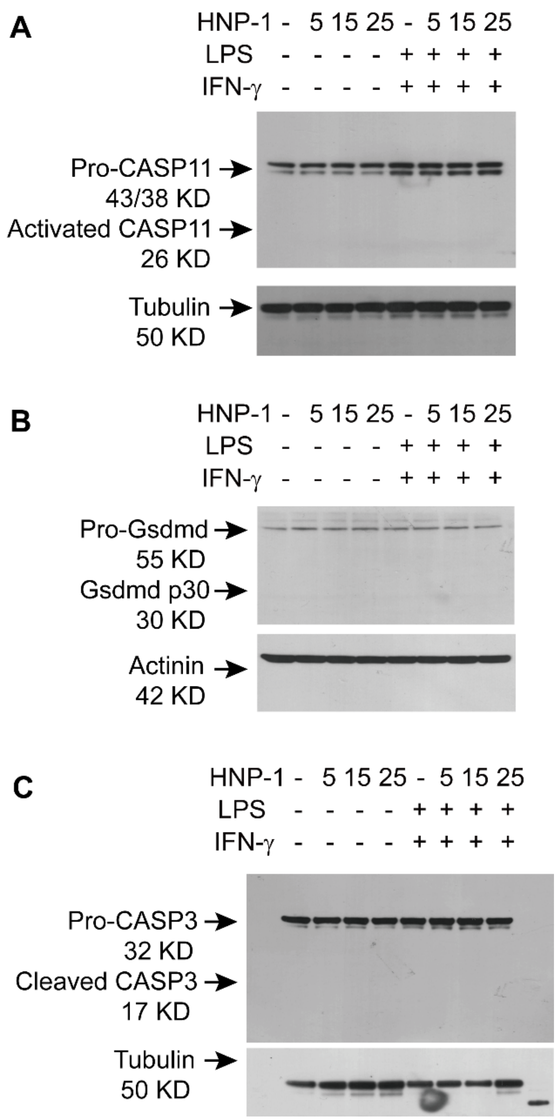

Figure S10. HNP-1-induced endothelial cell death does not involve caspase-11 and gasdermin D activation as well as caspase-3 activation. (*A*) Representative western blot analysis of caspase-11 precursor (Pro-CASP11) and activated caspase-11 (Activated CASP11) following exposure to different concentrations of HNP-1 (0 μg/ml, 5 μg/ml, 15 μg/ml, 25 μg/ml) in mouse lung microvascular endothelial cells (MLMECs) that were already primed with lipopolysaccharide (LPS; 1 μg/ml) and interferon-γ (IFN-γ; 100 ng/ml). One representative of four independent experiments is shown. α-tubulin served as a loading control. The images are uncropped. (*B*) Representative western blot analysis of gasdermin D precursor (Pro-Gsdmd) and activated gasdermin D (Gsdmd p30) following exposure to different concentrations of HNP-1 (0 μg/ml, 5 μg/ml, 15 μg/ml, 25 μg/ml) in MLMECs that were already primed with LPS (1 μg/ml) and IFN-γ (100 ng/ml). One representative of four independent experiments is shown. β-actin served as a loading control. The images are uncropped. (*C*) Representative western blot analysis of caspase-3 precursor (Pro-CASP3) and activated caspase-3 (Cleaved CASP3) following exposure to different concentrations of HNP-1 (0 μg/ml, 5 μg/ml, 15 μg/ml, 25 μg/ml) in MLMECs that were already primed with LPS (1 μg/ml) and IFN-γ (100 ng/ml). One representative of four independent experiments is shown. α-tubulin served as a loading control. The images are uncropped.

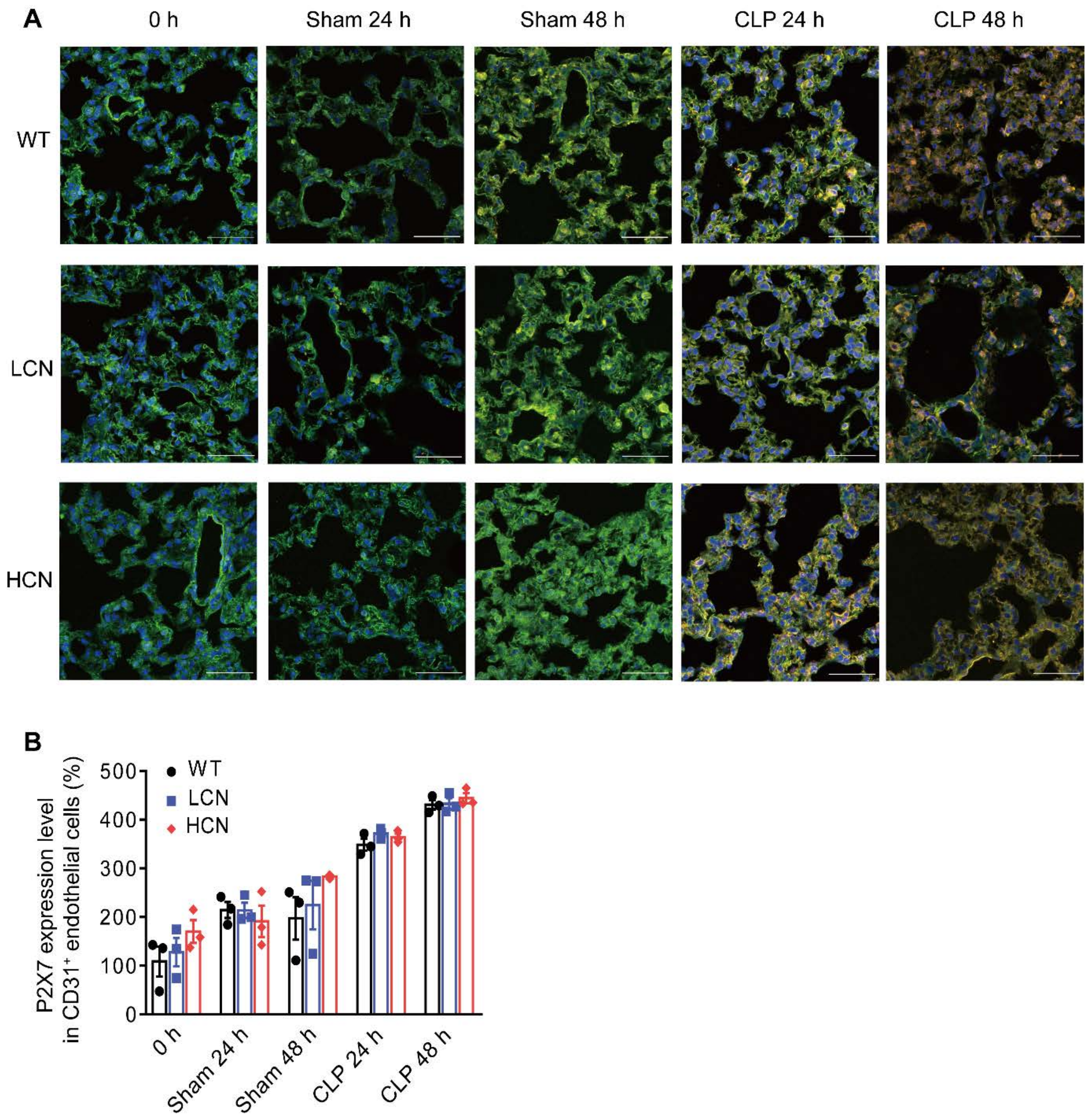

Figure S11. Expression of P2X7 in transgenic mice and WT mice after sepsis. Representative immunofluorescence images (*A*) and comparison of P2X7 expression (*B*) in the lungs of WT mice (*n* = 3 for each CLP and sham group), mice with LCN of *DEFA1/DEFA3* (*n* = 3 for each CLP and sham group), and mice with HCN of *DEFA1/DEFA3* (*n* = 3 for each CLP and sham group) at 0 h, and 24 h, 48 h after performance of CLP or sham operation. Red indicates P2X7 staining; green indicates CD31 staining; blue indicates DAPI staining. Scale bars, 50 μm. In *B*, data are shown as the mean ± s.e.m. using bar graphs overlaying the corresponding dot plots.

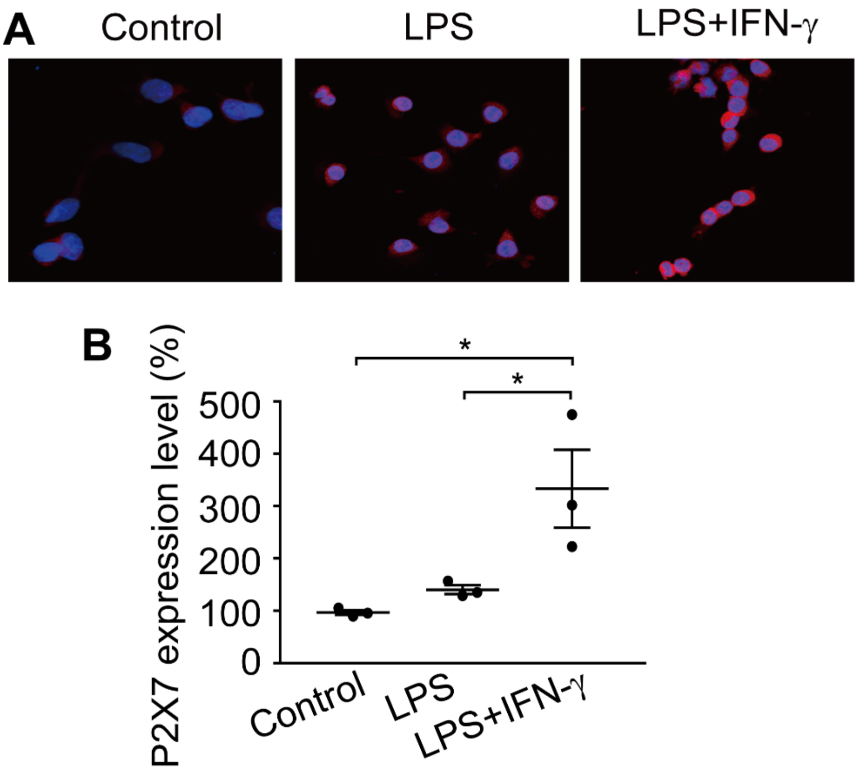

Figure S12. Increased expression level of P2X7 in stimulated mouse lung microvascular endothelial cells (MLMECs). Representative immunofluorescence images (*A*) and comparisons (*B*) of P2X7 expression in MLMECs that were unstimulated (control) or stimulated with lipopolysaccharide (LPS; 1 μg/ml) alone or with both LPS (1 μg/ml) and interferon-gamma (IFN-gamma; 100 ng/ml). One representative of three independent experiments is shown. Red indicates P2X7 staining; blue indicates DAPI staining. Scale bars, 50 μm. In *B*, data are presented as dot plots with horizontal bars representing means ± s.e.m.; *P = 0.017 between control group and LPS+IFN-γ group, *P = 0.039 between LPS group and LPS+IFN-γ group, one-way ANOVA with Bonferroni post tests.

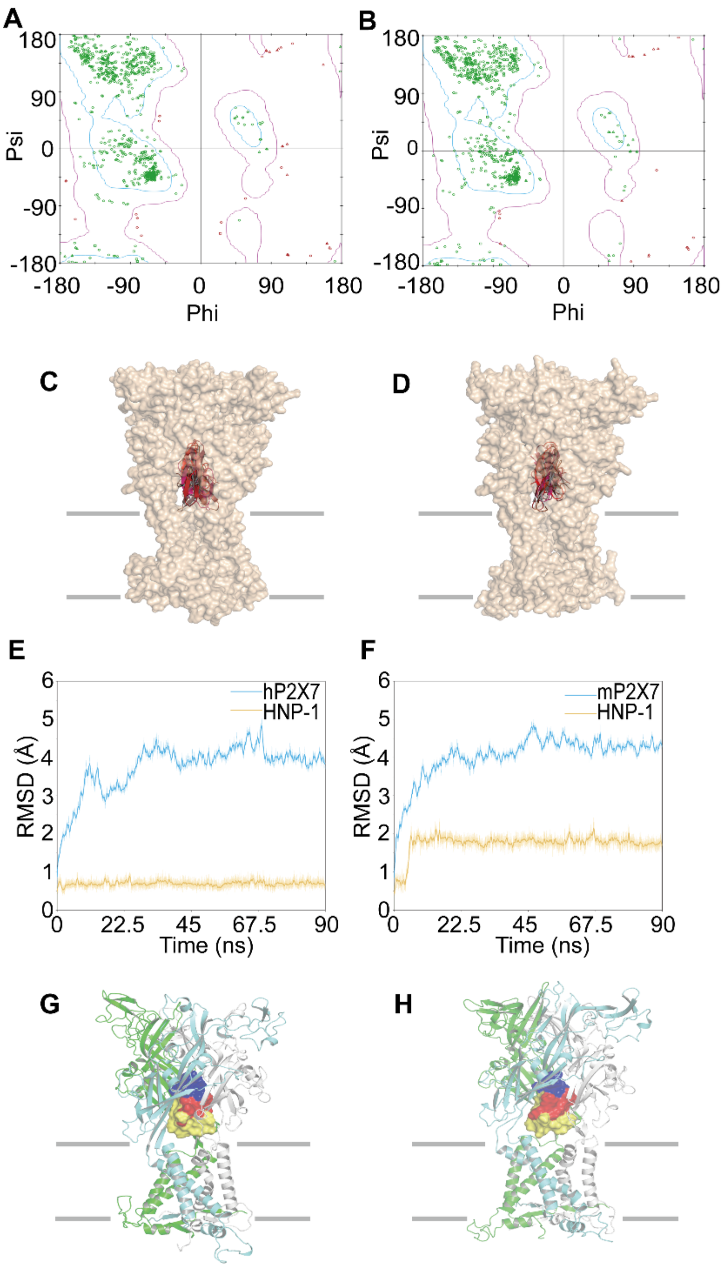

Figure S13. Modeling the interaction between HNP-1 and P2X7. (*A* and *B*) Ramachandran plot of the homology models of hP2X7 (*A*) and mP2X7 (*B*). Residues in the most favored regions, additional allowed regions, generously allowed regions, and disallowed regions are 86.5%, 11.8%, 1.0%, and 0.7%, respectively, for hP2X7, and 87.9%, 10.7%, 1.0%, and 0.4%, respectively, for mP2X7. (*C* and *D*) The top 10 ranked binding poses of HNP-1 in complex with hP2X7 (*C*) or mP2X7 (*D*). The three monomers of P2X7 are colored in wheat, and the top 10 ranked binding poses of HNP-1 are colored in red. (*E* and *F*) RMSDs of C_α atoms are shown as a function of time for HNP-1/hP2X7 (*E*) and HNP-1/mP2X7 (*F*). Time evolution of the RMSDs of P2X7 and HNP-1 are shown with blue and yellow lines, respectively. (*G* and *H*) Structure analysis of the last snapshot of the HNP-1/hP2X7 complex (*G*) or the HNP-1/mP2X7 complex (*H*) from the MD simulations. Cartoon views are presented. The three monomers of hP2X7 or mP2X7 are colored in green, cyan, and gray, respectively. The CYCRIPA motif, IYQGRLW motif and other motifs of HNP-1 are colored in red, blue, and yellow, respectively.

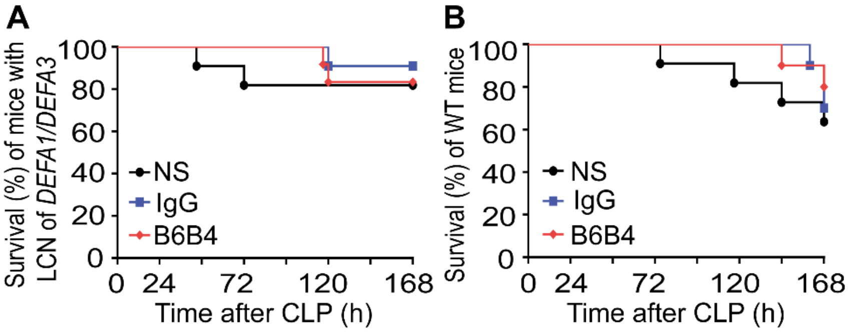

Figure S14. The blocking antibody B6B4 does not affect the survival of mice with LCN of *DEFA1/DEFA3* and WT mice after sepsis. Mice were subjected to CLP, and B6B4 (300 μg/mouse), mouse IgG (300 μg/mouse), or normal sodium (NS; equal volume) were intravenously administered at 0, 24 h and 48 h after CLP. Survival over time of mice with LCN of *DEFA1/DEFA3* (*A*) and WT mice (*B*) was assessed. In *A*, $n = 11$ for mice with NS treatment, $n = 11$ for mice with IgG treatment, and $n = 12$ for mice with B6B4 treatment. In *B*, $n = 11$ for mice with NS treatment, $n = 10$ for mice with IgG treatment, and $n = 10$ for mice with B6B4 treatment.

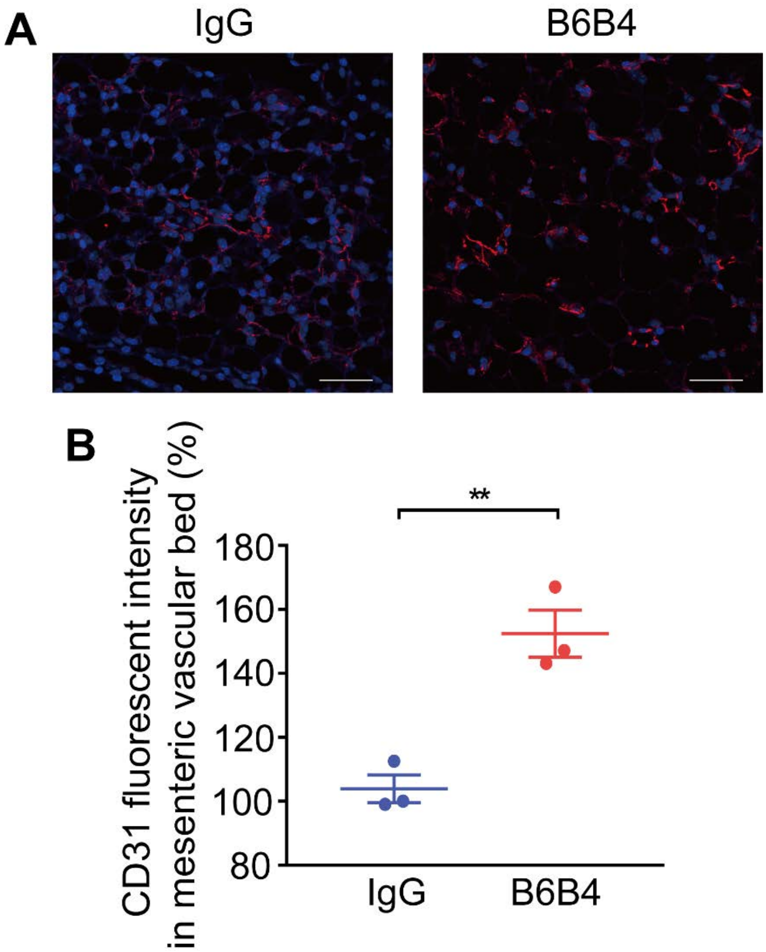

Figure S15. Treatment with the blocking antibody B6B4 increases CD31$^+$ endothelial cells in mesenteric vessels in mice with HCN of *DEFA1/DEFA3* after sepsis. Mice were subjected to CLP, and B6B4 (300 μg/mouse) and mouse IgG (300 μg/mouse) were intravenously administered at 12 h, 36 h and 60 h after CLP. Mice were killed at 72 h after CLP. Representative images (*A*) and comparisons (*B*) of CD31$^+$ endothelial cells in mesenteric vessels are shown (n = 3 for each group). Red indicates CD31$^+$ staining; blue indicates DAPI staining. Scale bars, 50 μm. In *B*, data are presented as dot plots with horizontal bars representing means ± s.e.m.; ***P* = 0.005, two-tailed unpaired *t* test.

Figure 4A

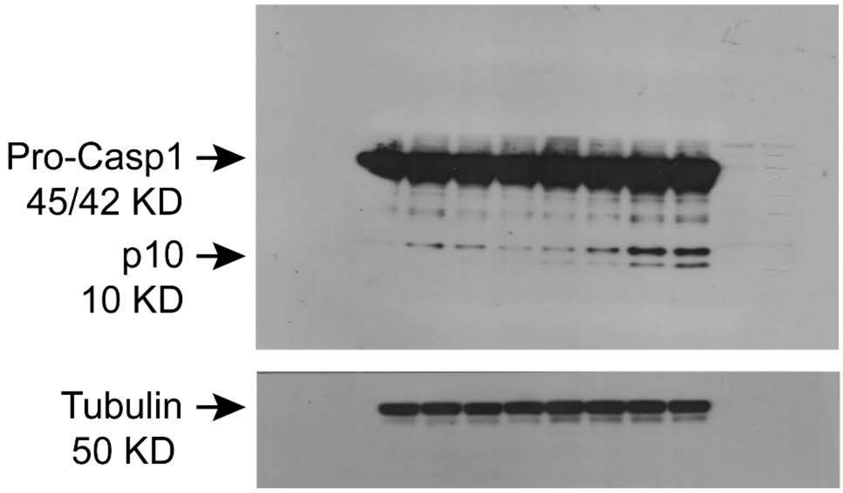

Figure 4C

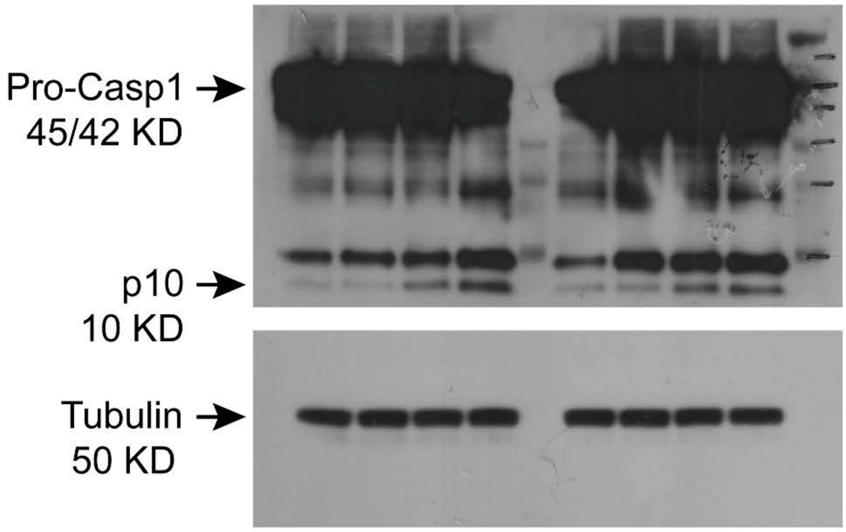

Figure 5E

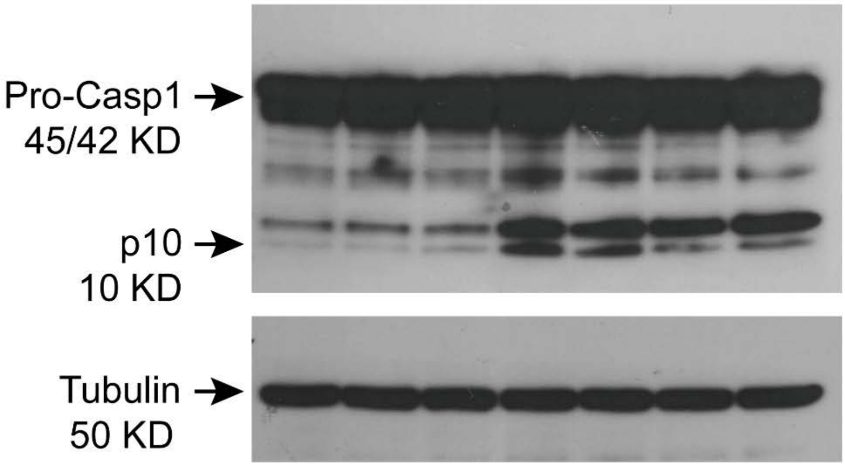

Figure S16. Uncropped images of the immunoblots for Figure 4*A*, Figure 4*C* and Figure 5*E*.

Movie S1. Time-lapse of leukocyte adhesion in WT mice at 72 h after CLP. Scale bar, 100 μm.

Movie S2. Time-lapse of leukocyte adhesion in mice with LCN of *DEFA1/DEFA3* at 72 h after CLP. Scale bar, 100 μm.

Movie S3. Time-lapse of leukocyte adhesion in mice with HCN of *DEFA1/DEFA3* at 72 h after CLP. Scale bar, 100 μm.

Movie S4. Time-lapse of leukocyte adhesion in WT mice at 72 h after sham operation. Scale bar, 100 μm.

Movie S5. Time-lapse of leukocyte adhesion in mice with LCN of *DEFA1/DEFA3* at 72 h after sham operation. Scale bar, 100 μm.

Movie S6. Time-lapse of leukocyte adhesion in mice with HCN of *DEFA1/DEFA3* at 72 h after sham operation. Scale bar, 100 μm.

Movie S7. Treatment with B6B4 rejuvenates septic mice with HCN of *DEFA1/DEFA3*.

References

1. Yue N, et al. (2017) Activation of P2X7 receptor and NLRP3 inflammasome assemblyin hippocampal glial cells mediates chronic stress-induced depressive-like behaviors. *J Neuroinflammation* 14:102.
2. Yao X, et al. (2017) Remodelling of the gut microbiota by hyperactive NLRP3 induces regulatory T cells to maintain homeostasis. *Nat Commun* 8:1896.
3. Eckle T, Grenz A, Laucher S, Eltzschig HK (2008) A2B adenosine receptor signaling attenuates acute lung injury by enhancing alveolar fluid clearance in mice. *J Clin Invest* 118:3301-3315.
4. He S, et al. (2009) A complement- dependent balance between hepatic ischemia/reperfusion injury and liver regeneration in mice. *J Clin Invest* 119:2304-2316.
5. Ismail OZ, et al. (2015) Kidney injury molecule-1 protects against G-α12 activation and tissue damage in renal ischemia-reperfusion injury. *Am J Pathol* 185:1207-1215.
6. Arai S, et al. (2016) Apoptosis inhibitor of macrophage protein enhances intraluminal debris clearance and ameliorates acute kidney injury in mice. *Nat Med* 22:183-193.
7. Standiford TJ, et al. (1995) Macrophage inflammatory protein-1 alpha mediates lung leukocyte recruitment, lung capillary leak, and early mortality in murine endotoxemia. *J Immunol* 155:1515-1524.
8. Bdeir K, et al. (2010) Neutrophil alpha-defensins cause lung injury by disrupting the capillary-epithelial barrier. *Am J Respir Crit Care Med* 181:935-946.
